# Analysis of a Marseillevirus Transcriptome Reveals Temporal Gene Expression Profile and Host Transcriptional Shift

**DOI:** 10.3389/fmicb.2020.00651

**Published:** 2020-04-14

**Authors:** Rodrigo Araújo Lima Rodrigues, Amina Cherif Louazani, Agnello Picorelli, Graziele Pereira Oliveira, Francisco Pereira Lobo, Philippe Colson, Bernard La Scola, Jônatas Santos Abrahão

**Affiliations:** ^1^Laboratório de Vírus, Departamento de Microbiologia, Instituto de Ciências Biológicas, Universidade Federal de Minas Gerais, Belo Horizonte, Brazil; ^2^Microbes, Evolution, Phylogeny and Infection (MEΦI), IRD 198, Assistance Publique-Hopitaux de Marseille (AP-HM), Aix-Marseille Université UM63, Marseille, France; ^3^Laboratório de Algoritmos em Biologia, Departamento de Genética, Ecologia e Evolução, Instituto de Ciências Biológicas, Universidade Federal de Minas Gerais, Belo Horizonte, Brazil; ^4^Institut Hospitalo-Universitaire (IHU) - Méditerranée Infection, Marseille, France

**Keywords:** giant virus, marseillevirus, transcriptome, RNA-seq, gene expression, promoter motifs

## Abstract

Marseilleviruses comprise a family of large double-stranded DNA viruses belonging to the proposed order “Megavirales.” These viruses have a circular genome of ∼370 kbp, coding hundreds of genes. Over a half of their genes are associated with AT-rich putative promoter motifs, which have been demonstrated to be important for gene regulation. However, the transcriptional profile of Marseilleviruses is currently unknown. Here we used RNA sequencing technology to get a general transcriptional profile of Marseilleviruses. Eight million 75-bp-long nucleotide sequences were robustly mapped to all 457 genes initially predicted for Marseillevirus isolate T19, the prototype strain of the family, and we were able to assemble 359 viral contigs using a genome-guided approach with stringent parameters. These reads were differentially mapped to the genes according to the replicative cycle time point from which they were obtained. Cluster analysis indicated the existence of three main temporal categories of gene expression, early, intermediate and late, which were validated by quantitative reverse transcription polymerase chain reaction assays targeting several genes. Genes belonging to different functional groups exhibited distinct expression levels throughout the infection cycle. We observed that the previously predicted promoter motif, AAATATTT, as well as new predicted motifs, were not specifically related to any of the temporal or functional classes of genes, suggesting that other components are involved in temporally regulating virus transcription. Moreover, the host transcription machinery is heavily altered, and many genes are down regulated, including those related to translation process. This study provides an overview of the transcriptional landscape of Marseilleviruses.

## Introduction

The family *Marseilleviridae* is a recently established taxon encompassing Marseilleviruses, an unusual group of giant viruses isolated mostly from water samples, by co-culturing on *Acanthamoeba* spp. (Boyer et al., 2009; Colson et al., 2013b). This virus family has been expanding over the last few years, with isolates from different regions around the world, such as Senegal, Tunisia, India, Japan, Australia, Brazil, and New Caledonia (Lagier et al., 2012; Aherfi et al., 2014; Doutre et al., 2014; Dornas et al., 2016; Takemura, 2016; Chatterjee and Kondabagil, 2017; Fabre et al., 2017). Along with other giant viruses isolated on amoebae, the Marseilleviruses are members of the proposed order “Megavirales,” which comprises the nucleocytoplasmic large DNA viruses (NCLDVs) (Colson et al., 2013a). They replicate in free-living amoebae of the genus *Acanthamoeba*. The replication cycle begins with phagocytosis of multiple viral particles, with membranous vesicles containing thousands of particles within 1 h of infection (Arantes et al., 2016). Once within the host cells, the Marseilleviruses establish large cellular structures (factories) for progeny virus production around 3 to 4 h post-infection (p.i.), with genome replication being assisted by host nuclear proteins, morphogenesis occurs, and the viral progeny is released by cell lysis or wrapped inside giant infectious vesicles 8 h after infection (Arantes et al., 2016; Fabre et al., 2017).

Marseillevirus T19 (MRSV) was the first virus to be described in this group, the prototype of the genus *Marseillevirus*, family *Marseilleviridae*. It has an icosahedral capsid of 250 nm diameter and a circular double-strand DNA genome of 368 kb, containing 457 open reading frames (ORFs) predicted to encode proteins, the large majority of which have no known function (Boyer et al., 2009). The genomes of Marseilleviruses exhibit a high degree of mosaicism, with genes having different origins (Boyer et al., 2009). Over a half of these genes are associated with a promoter motif, AAATATTT, which has been shown to be important in driving gene expression *in vitro* and is present as single or multiple copies in intergenic regions (Oliveira et al., 2017b). AT-rich promoter motifs have been predicted for other amoebal giant viruses, such as mimiviruses, faustoviruses and kaumoebavirus, where they seem to be associated with temporal regulation of viral gene expression throughout the infection cycle of some viruses (Legendre et al., 2010; Oliveira et al., 2018). Currently, there is no information about the transcriptome of Marseilleviruses, and which of the predicted genes are expressed during viral replication in its amoebae host. This represents important information in better understanding the biology of these viruses.

In this work we aimed to obtain information on temporal patterns of gene expression for the Marseillevirus prototype isolate and the impact on host transcriptional activity by viral infection. Data was obtained by RNA sequencing (RNA-seq) and validated by quantitative reverse transcription polymerase chain reactions (RT-qPCR). This work provided an overview of the transcriptional profile of Marseilleviruses-host interaction, expanding our knowledge about the biology of these viruses.

## Materials and Methods

### Cell Culture and Infection of Marseillevirus

For virus production, *Acanthamoeba castellanii* strain Neff (ATCC 30010) cells were infected with MRSV and cultivated in T150 cm^2^ cell culture flasks containing peptone-yeast extract-glucose (PYG) medium, at 30°C for up to 7 days. After complete cell lysis, the material was collected and passed through a 0.45 μm membranous filter (Millipore, United States) to remove cell debris. The virus was titrated by the end-point method, with the titers being expressed as 50% tissue culture infectious doses (TCID_50_) (Reed and Muench, 1938), and was refrigerated until further use.

For transcriptome analysis, a total of 9 infection points was assayed, 0, 1, 2, 4, 5, 6, 8, 10, and 12 h p.i., based on the replication profile observed for the virus during one-step growth curve assays (Supplementary Figure S1). T150 cm^2^ cell culture flasks, each containing 2 × 10^7^ cells of *A. castellanii* in 25 mL of PYG medium, were infected with MRSV at a multiplicity of infection (MOI) of 100. The cells were left at 30°C for 30 min, and then the supernatant was removed and the cell monolayer was washed twice with Page’s Amoeba Saline buffer to remove excess virus. Fresh PYG medium was added in each flask, which was then incubated at 30°C, with cells being collected at each designated time point. The time point of 0 h corresponded to 30 min p.i., representing the adsorption period. The samples were collected and centrifuged for 10 min at 1,000 × *g*. Supernatant was discarded and the cell pellet resuspended in RNA*later* stabilization reagent (QIAgen, France) and kept at −20°C until further use.

### RNA Extraction and Ribosomal RNA Depletion

RNA extraction was performed using the RNeasy Midi Kit (QIAgen, France), following the manufacturer’s protocol. Briefly, cells were centrifuged for 10 min at 3,000 × *g* to remove the RNA*later* reagent and then resuspended in 4 mL RLT buffer for cell lysis. On-column DNA digestion was performed. The total RNA was then eluted with two successive additions of ∼200 μL RNase free water. Then, two further digestions with Turbo DNase (Invitrogen, United States) were performed for each RNA sample to eliminate DNA contamination. Each treatment involved incubating the samples with Turbo DNase for 30 min at 37°C. Finally, enzyme inactivation reagent was added and incubated at room temperature for 5 min. The samples were then centrifuged at 10,000 × g for 1.5 min, collected and quantified with a NanoDrop spectrophotometer. The absence of contaminating DNA was checked by quantitative PCR using the following primers: Fwd 5′-TCTGGGAGTGGGCTTTATCT-3′; Rev 5′-AGGGTAATGACCTCGGGTA-3′ (amplicon size of 183 bp).

For mRNA enrichment, a crucial step before RNA sequencing, the ribosomal RNA (rRNA) depletion strategy was performed using the Ribo-Zero rRNA removal Kit (Bacteria; Illumina, France), following the manufacturer’s protocol. Briefly, 2.5 μg of each RNA sample was hybridized with probes for rRNA, incubated at 68°C for 10 min, and then associated to magnetic beads for rRNA removal (incubation at 50°C for 5 min). RNA was then purified by ethanol precipitation, resuspended in the specific buffer for sequencing library preparation, and the ribosomal depletion was checked by using an Agilent 2100 Bioanalyzer with an RNA 6000 Pico Chip (Supplementary Figure S2). The depleted RNAs were used for construction of the sequencing libraries.

### cDNA Production and Sequencing

cDNA synthesis and library construction for sequencing was performed using the TruSeq stranded total RNA kit (Illumina, France), following the manufacturer’s protocol. Briefly, first strand cDNA synthesis was performed with SuperScript II enzyme. After second strand synthesis, cDNAs were adenylated at their 3′ ends, adaptors were added, and PCR amplification was performed to obtain the library. Each library profile was visualized on a DNA 1000 Bioanalyzer LabChip (Agilent Technologies Inc., United States) to determine the optimum size in base-pairs, with the final library concentration measured in nmol/l. Libraries were normalized at 2 nM and pooled for sequencing using MiSeq Reagent kit V3 at 150 cycles.

### Sequence Read Mapping, Assignment and Count

Nucleotide sequence reads generated from each RNA-seq dataset were uploaded to the Galaxy web platform, and the public server at http://usegalaxy.org was used for part of the data analysis (Afgan et al., 2018). Reads were mapped on the Marseillevirus T19 genome (GenBank accession number NC_013756.1) using HISAT2 software (Kim et al., 2015) with default parameters (see entire protocol in Supplementary Figure S3), and considering a maximum intron length of 5000 bp as previously described (Cherif Louazani et al., 2018). Reads that did not align to the viral genome were mapped against *Acanthamoeba castellanii Neff* nuclear genome (GenBank accession number AHJI00000000.1) using HISAT2 software default parameters adapted to eukaryotic genomes with maximum intron length set to 500 000 bp and excluding all discordant alignment (Kim et al., 2015). Unaligned reads at this step were then mapped against the amoeba mitochondrial genome (GenBank accession number NC_001637.1) using similar parameters only differing in maximum intron length, this time set to 1000 bp to correspond to the mitochondrial genome organization (Burger et al., 1995). Mapping results were analyzed using HTseq-count software, with the union mode (Anders et al., 2015). Only uniquely mapped reads were used in further analyses.

### Marseillevirus Reads Assembly

To validate gene structures of Marseillevirus, viral reads of all infection time points were pooled then assembled into contigs with Trinity genome-guided approach using jaccard-clip and trimmomatic options (Grabherr et al., 2011). Contigs were then mapped against the viral genome using PASA pipeline with stringent overlap parameters (Haas et al., 2003). Contigs were then inspected for ORFs using Transdecoder (Haas et al., 2013). Predicted ORFs of over 100 aa were aligned against the annotated MRSV proteins using protein BLAST (Altschul et al., 1997). Predicted ORF that did not align to known MRSV proteins were searched for homologs against the NCBI non-redundant protein sequences database and inspected for functional domains using the NCBI Conserved Domain Database search tool. In parallel, to correct genome structure Marseillevirus genes that were predicted to lack valid start codon were aligned against the assembled contigs database using nucleotide BLAST (Zhang et al., 2000). Contigs were considered valid transcripts if they matched a predicted viral protein or gene with a minimum overlap of 70%.

### Marseillevirus Gene Expression Count Normalization and Cluster Analysis

For Marseillevirus expression analysis, the raw counts were normalized, considering gene length and sequencing coverage, by means of Transcripts Per Million reads (TPM) (Conesa et al., 2016). Normalized read counts ranged from 32 to 318,169. To reveal transcriptional patterns during Marseillevirus infection, we clustered gene transcription profiles using hierarchical and k-means clustering methods. We first log-transformed the normalized read count profiles and centered this data by the mean. Both cluster analyses were performed with the Cluster 3.0 program (de Hoon et al., 2004), using Euclidean distance as the similarity metric, and setting the number of clusters to 3. Data visualization was done using the Java TreeView program (Saldanha, 2004).

### Expression Profile Validation by Real-Time Quantitative PCR

To validate the expression profiles observed with the RNA-seq analysis, we performed reverse transcription real-time polymerase chain reactions (RT-qPCR) for six different genes (Supplementary Table S1). We included two genes with high level of expression from each temporal category defined in the RNA-seq data for this assay. Specific primers for each gene were designed using the primer-blast tool on the National Center for Biotechnology Information platform^1^. Virus infection and RNA extraction was performed as previously described. RT-qPCR was performed in a one-step reaction using the QuantiTect SYBR Green RT-PCR Kit (QIAgen, France), following the manufacturer’s recommendations. Assays were performed in a Bio-Rad Real-Time PCR Detection System (Bio-Rad) using the following thermal conditions for all genes, 30 min at 50°C for the reverse transcription step, followed by 15 min at 95°C and 40 amplification cycles of 15 s at 94°C, 30 s at 60°C, and 30 s at 72°C. Assay values were expressed as arbitrary units (delta-Ct). For each assay, the experiment was performed twice.

### *De novo* Motif Search and Statistical Analysis

We used MEME (Motif Discovery Tool, version 5.0.4 (Bailey et al., 2009) for *de novo* motif prediction in the intergenic sequences of MRSV. For that purpose, we used a fasta formatted file containing all the 426 intergenic regions sequences (size cutoff = 8 nucleotides), where each sequence corresponds to the upstream sequence from the strand where the gene was observed. This fasta file was used as input to a stand-alone version of MEME running on a local Dell Server (2 Intel Xeon E5-4610 v2 2.3GHz processors, 64 threads, 128GB RAM, CentOS Linux release 7.5.1804). We executed MEME in parallel (60 threads) with the following configuration: (1) motif length varying between 8 and 10 nucleotides (an additional search was performed considering motif length varying between 5 and 15 nucleotides); (2) search for zero or more motifs in each intergenic region; (3) no upper limit for the number of sequences per intergenic region. The command line used to generate the results is reported below:

command: meme in_file.fasta -dna -o meme_output_file.txt -csites 5000 -mod anr -nmotifs 10 -minw 8 -maxw 10 -p 60

All graphs and statistical analyses reported here were done using R and Python (McKinney, 2010; Wickham, 2016). We developed Python scripts to parse the MEME output file filtering each motif occurrence reported based on its, respectively e-value into a dataframe using the Pandas and Numpy packages (Van Der Walt et al., 2011). We have selected the best four ranked motifs for forward analyses in R environment. We used additional software for *de novo* motif prediction, the BaMMmotif server (Kiesel et al., 2018), in order to validate the results found in MEME search.

### Host Response to Infection

Amoebal nuclear genes were analyzed for differential expression using EdgeR. First, raw read counts were normalized for library sizes with the trimmed mean of M-values (TMM) method (Robinson et al., 2010). A preliminary principal component analysis showed a clustering of samples according to the infection time into 3 groups: early group (0, 1, and 2 h p.i.), intermediate group (4, 5, 6, and 8 h p.i.) and late group (10 and 12 h p.i.). Using EdgeR quasi-likelihood test between the three groups of samples with robust settings, the expression of 48 genes was estimated significantly altered at a False Discovery Rate of 0.05 and a log2-fold-change of at least 1. Amongst these differentially expressed genes, 29 showed an overall decrease in their expression level in the course of infection while 19 showed an increase in expression. The functional annotation of differentially expressed genes was conducted using BlastKOALA (Kanehisa et al., 2016) and Amoebadb (Aurrecoechea et al., 2011).

Parallelly, the expression of the amoeba mitochondrial genes was inspected for a potential temporal transcriptional pattern. After the normalization of raw read counts for gene length and library size with the Transcripts Per Million reads method (TPM) (Conesa et al., 2016), a principal component analysis was performed on the nine samples. In absence of clustering of the samples by time of infection, normalized read counts were log-transformed and genes were clustered using Euclidean distance and average linkage. Data clustering and visualization were performed using ClustVis webtool (Metsalu and Vilo, 2015). The functional classification of mitochondrial genes followed the results of Gawryluk et al. (2014) proteomic and bioinformatic study.

## Results

### Predicted Genes of the Marseillevirus Were Validated by RNA-Seq Data

A ribosomal depletion approach was used for preparation of the RNA to be sequenced. *Acanthamoeba castellanii* strain Neff was infected with MRSV, and cells were collected at different times. Nucleotide sequencing datasets obtained from these separate cell collections for times p.i. of 0, 1, 2, 4, 5, 6, 8, 10, and 12 h, were mapped to the virus genome. All 457 initially predicted genes of MRSV (Boyer et al., 2009) were validated by at least 10 reads superimposed on the corresponding ORF (considering raw data from all time-point datasets). The total number of reads normalized by means of transcripts per million reads (TPM) exhibited a highly differential distribution among the genes (min = 32; max = 318,169; median = 7,036), indicating a large difference in the expression level of the respective genes (Supplementary Figure S4 and Supplementary Table S2). Indeed, an initial analysis of the 20 most expressed genes suggested differential gene expression throughout the viral replication cycle, wherein some genes had more cognate reads in earlier (e.g., MAR_ORF412 and MAR_ORF014) or later periods (e.g., MAR_ORF300 and MAR_ORF342) (Table 1 and Supplementary Table S3). Among these most expressed genes, only 4 had predicted functions, one of them being the major capsid protein gene. We performed additional *in silico* analysis attempting to gain insights about the function of these highly expressed genes. Only two genes previously annotated as hypothetical protein, i.e., with unknown function, had a conserved protein domain fold identified, suggesting peptidase activity (MAR_ORF026) and dehydrogenase activity (MAR_ORF021) (Supplementary Table S4). The fact that the majority of the most expressed genes of MRSV have no known function was intriguing and deserves to be further studied, since they were presumably highly important genes in the virus life cycle.

**TABLE 1:** The top 20 most expressed annotated genes.

Gene	Presence of promoter	Expression pattern	Predicted function	0 h	1 h	2 h	4 h	5 h	6 h	8 h	10 h	12 h	Total
MAR_ORF300	N	L	–	1349	31	69	36587	49038	50252	55730	67099	58015	318169
MAR_ORF390	Y	L	multiple zinc ribbon protein	1776	9869	27205	51234	49629	43289	39411	45095	50282	317790
MAR_ORF317	N	L	–	1354	170	40	36306	39576	39271	54230	35973	68786	275705
MAR_ORF342	Y	L	major capsid protein	609	48	26	61325	45875	40048	66725	23909	30871	269436
MAR_ORF384	N	L	–	899	134	23	38560	35602	42222	50020	54510	34821	256790
MAR_ORF370	N	L	–	1317	317	948	17200	28825	27160	39832	89992	36077	241669
MAR_ORF219	Y	L	–	793	106	12744	84839	47325	22149	20213	17578	19583	225329
MAR_ORF029	N	I	–	1843	24117	60761	27563	19259	17693	24533	14184	22469	212422
MAR_ORF305	Y	L	–	379	32	28	35828	39222	43383	28499	15090	25099	187560
MAR_ORF413	Y	L	histone H3	520	44	66	16092	35578	41292	29851	22200	25556	171199
MAR_ORF109	Y	L	–	926	260	24676	29570	17876	9340	5808	5875	10734	105065
MAR_ORF421	N	L	–	210	62	1986	17949	17338	21450	21452	9317	15270	105033
MAR_ORF412	N	E	–	54414	40209	5914	197	101	196	130	202	192	101555
MAR_ORF026	Y	L	–	402	179	173	6830	15641	19574	15741	14843	27536	100920
MAR_ORF193	Y	L	–	385	74	2351	13831	16722	14791	16327	15747	17850	98078
MAR_ORF021	Y	L	zinc finger protein	720	77	16	8775	16500	19814	19344	15478	17237	97962
MAR_ORF389	Y	E	–	27733	43921	21364	514	409	534	396	744	438	96053
MAR_ORF250	N	L	–	202	227	94	15714	20837	21501	16581	9799	10734	95689
MAR_ORF014	N	E	–	66374	22286	2559	98	84	17	59	53	62	91592
MAR_ORF147	N	E	–	44432	36392	2414	141	94	117	85	77	58	83810

*The total number of normalized reads counts is presented for each gene as well as the read count at each time point of infection. N, no; Y, yes; E, early; I, intermediate; L, late.*

To further investigate the structure of MRSV genes, RNA-seq reads were assembled into contigs using a genome-guided approach. Considering the predicted coding density of MRSV genome and to avoid transcripts overlapping neighboring genes with short UTRs, stringent overlap parameters were applied. This analysis allowed the assembly of 359 contigs. A first validation step considered protein coding genes only, contigs were therefore inspected for Open Reading Frames and their corresponding protein sequences were looked for homology to known MRSV proteins. This step led to the validation of 122 contigs. Predicted genes of MRSV lacking valid start codon were considered in the second validation step. An alignment of these genes with the assembled transcripts database helped validate seven additional contigs. These contigs confirmed the genome sequence of the predicted genes lacking start codon as they presented 100% identity with the genomic sequence. The last validation step inspected the presence of potential novel protein coding genes and non-coding transcripts. For this, assembled contigs that did not match known Marseillevirus genes were searched for homology at the nucleotide and protein level. In total, the read assembly allowed the definition of the 3-prime UTRs of 75 genes, the 5-prime UTRs of 27 genes and internal transcripts corresponding to 44 predicted MRSV genes (Supplementary Table S5).

### Transcriptional Profile of Marseillevirus Genes

**FIGURE 1:**
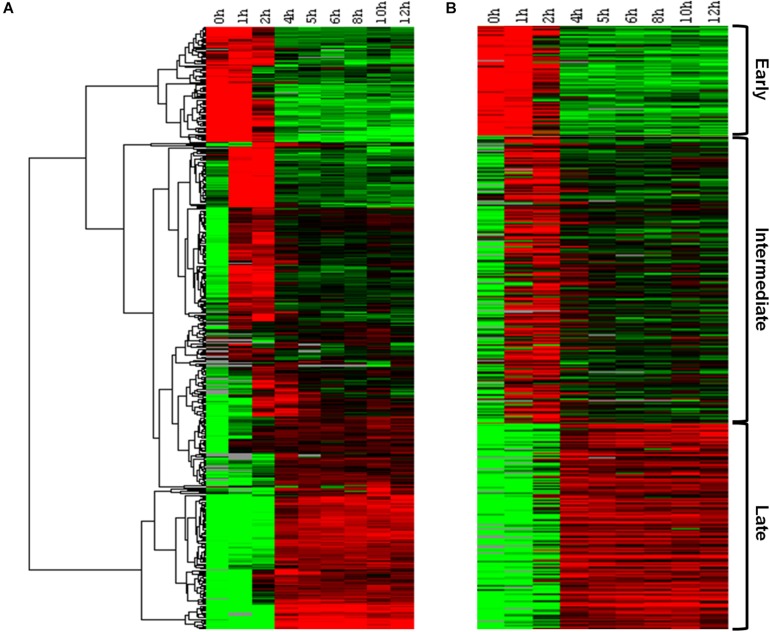
Marseillevirus gene expression classes. **(A)** Heat map of Marseillevirus gene expression profiles. Rows correspond to all 457 putative genes and columns to the 9 infection time points (0 h corresponds to 30 min of infection due to virus adsorption period). Expression profiles are clustered using hierarchical clustering. A dendrogram of the clustering is shown on the left. **(B)** Heat map of the same expression profiles partitioned into three main classes, “early” (top), “intermediate” (center), and “late” (bottom), with the k-means clustering algorithm. Expression levels are displayed from green (low expression) to red (high expression). Gray lines indicate absence of data for the gene at a particular infection time point. Both clustering methods were performed by applying Euclidean distance as a similarity metric. Among the 457 MRSV-encoded genes, 83 (18%) are expressed early, 218 (48%) are intermediate genes, and 156 (34%) are late genes. The function of each gene related to their temporal expression pattern can be found in Supplementary Data Sheet S1.

In order to confirm the existence of a temporal pattern of gene expression for Marseilleviruses, traditional hierarchical cluster analysis was initially performed without any prior information, allowing the grouping of genes that share similar expression profiles. This analysis indicated the existence of three main groups, comprising: (i) genes for which expression levels were high 0–1 h p.i., remained slightly elevated to 2 h p.i., and then decreased; (ii) genes with low expression at 0 h, that are expressed 1–2 h p.i., and also during later periods of the cycle; and (iii) genes with high expression levels after 4 h p.i. and which remain detectable for at least 12 h p.i. (Figure 1A). This initial analysis demonstrated that not only were all predicted MRSV genes expressed, but that their expression was remarkably fast, occurring within 4 h p.i., with many of them remaining active until the stage of viral progeny release. A second cluster analysis, this time using the *k*-means strategy, where *k* was set to 3, confirmed the presence of three categories of genes, consistent with the traditional temporal categories of “early,” “intermediate,” and “late” virus genes, as observed in the hierarchical clustering analysis (Figure 1B). Our data showed that of the 457 MRSV-encoded genes, 83 (18%) were expressed early at 0–1 h p.i., 218 (48%) were intermediate genes with an expression peak at 1–2 h p.i., and 156 (34%) were late genes, with peak expression from 4 h p.i. (Supplementary Data Sheet S1).

**FIGURE 2:**
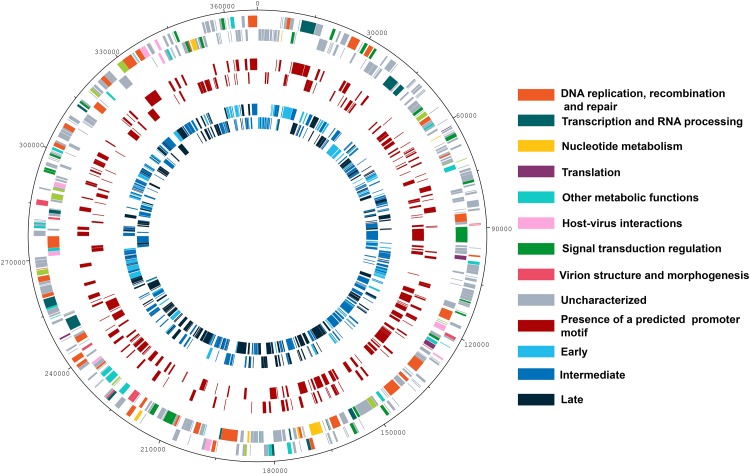
Genome map of Marseillevirus. Genome map highlighting the different categories of genes. Rings starting from outer to innermost correspond to (i) genome coordinates in kilobases; (ii) predicted protein-coding genes oriented forward or reverse on DNA strands, with different colors corresponding to distinct functional gene categories; (iii) genes followed by the predicted core AAATATTT promoter motif; (iv) distribution of genes from different temporal classes, early, intermediate, and late. A color legend is provided to the right of the figure.

Since data obtained from high throughput sequencing can contain some bias, leading to erroneous interpretations about the existence of temporal profiles of gene expression for Marseilleviruses, we also evaluated the presence of the three categories by means of RT-qPCR, a more sensitive technique and the golden standard method in gene expression analysis (Bustin, 2000; Bustin et al., 2005). Using six different genes, we confirmed the existence of the three classes of gene expression during the replication cycle of Marseilleviruses (Supplementary Figure S5). Early genes exhibited high expression level at 0 h and had an activation peak 1 h p.i., with a considerably reduced expression after this period. Intermediate genes had an increase in their expression levels at 1 h and reach their highest levels at 2 h p.i. In contrast with the early genes, their expression levels seemed to decrease more slowly after 4 h p.i., which suggested maintenance of their mRNAs in the host cell. Lastly, the late genes had an expression peak at 4 h p.i., and their expression was maintained over the remaining stages of viral replication. The expression peaks of the tested genes in RT-qPCR corresponded exactly to the three different temporal classes of genes identified by RNA-seq approach. As an internal control, we also observed whether there is a correlation between proteins found in MRSV particles (Boyer et al., 2009) and late expressed genes. All but six proteins found in viral particles are products from late expression genes; the other six proteins are products from intermediate expression genes (Supplementary Data Sheet S1). Genes belonging to the different temporal expression classes were homogeneously distributed around the viral genome. As such, structural clusters of genes in the genome based on their transcriptional profiles could not be observed (Figure 2).

### Functional Categories of Genes Based on Time Post-Infection

**FIGURE 3:**
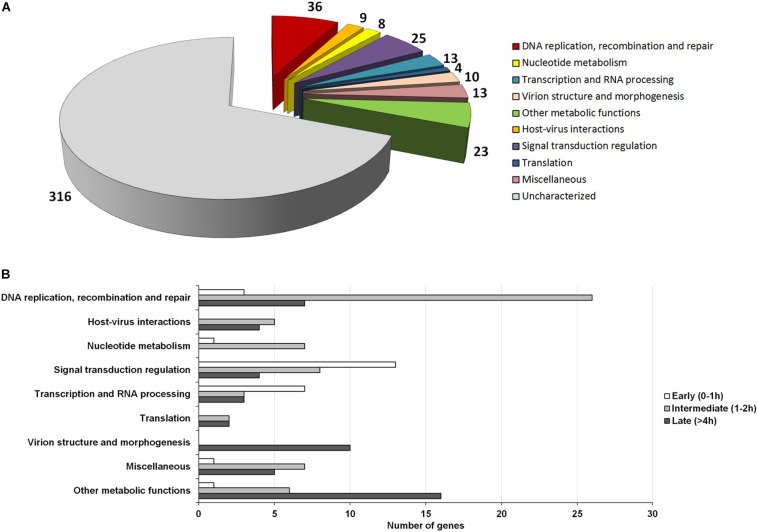
Functional category analysis and correlation to the temporal expression profile of viral genes. **(A)** Pie-chart representing the number of Marseillevirus genes distributed into different functional categories, according to the NCVOG clusters; **(B)** Distribution of genes from different functional categories into temporal gene expression classes. Only genes with known functions are included in the graph.

The NCLDVs were believed to share an intrinsic evolutionary history as a monophyletic group of viruses, having many homologous genes divided in different functional groups, the nucleo-cytoplasmic virus orthologous groups (NCVOGs) (Yutin et al., 2009). Based on this functional classification, 316 genes (69.1%) of MRSV had uncharacterized function, and the remaining genes were divided into nine categories, among which 36 genes were related to DNA replication, repair and recombination and 25 genes were related to signal transduction regulation. Among the other genes were those related to nucleotide metabolism (8 genes), transcription (13 genes), translation (4 genes), and viral structure and morphogenesis (10 genes) (Figure 3A). However, genes were not clustered on the genome based on these functional classifications (Figure 2). These groups comprised genes with differential expression levels throughout the virus replication cycle, although some categories (e.g., viral structure and morphogenesis) had genes that were only expressed during later periods, as expected (Figure 3B).

Among the genes related to DNA replication, repair and recombination, the majority were intermediate and late genes, although some genes were expressed as soon as the virus entered the host cells, such as alkylated DNA repair protein and XRN1 5′-3′ exonuclease genes (Figure 3B and Supplementary Figure S6). Some genes, including DNA polymerase B and DNA topoisomerase II genes were expressed 2 h p.i. Moreover, some genes, such as D6/D11 helicase and D5-primase-helicase exhibited late activation peaks (at 4 h p.i.) when the virus production was fully established (Supplementary Figure S6), which could be used and possibly incorporated into virus particles, as previously observed for other D6-helicases and DNA polymerases (Boyer et al., 2009; Fabre et al., 2017). Notably, restriction enzymes encoded by MRSV, which are important proteins in virus-host interactions, were expressed 2 h p.i., suggesting their participation prior to the establishment of cellular structures (factories) for virus production. The viral-encoded histones exhibited a late expression peak, indicating their direct role in final steps of the synthesis of viral nucleic acid, as previously suggested (Boyer et al., 2009; Thomas et al., 2011). It is remarkable that histone-like protein encoding genes are unique to MRSV among NCLDV and exhibit high level of expression, the histone H3 being one of the most expressed viral genes (Table 1). With respect to the genes involved in nucleotide metabolism, the great majority were expressed at intermediate points in the cycle, with peak expression within 2 h p.i., such as for ribonucleotide reductase and thymidine kinase genes (Figure 3B and Supplementary Figure S6).

Marseillevirus T19 had a large set of genes involved in signal transduction regulation, especially members of the serine/threonine (ST) kinase family, which suggested that the virus had the potential to manipulate the host response against infection (Bonjardim, 2017; Meineke et al., 2019). Most of these genes (13/25 = 52%) were expressed early in infection at 0 h p.i. (30 min after interaction with host cells) (Figure 3B). This fast expression profile suggested that the virus interfered with host response as soon as they made contact, which could facilitate the establishment of a productive infectious cycle. In addition, some genes were expressed later, after 4 h p.i., indicating that such regulation of host responses was maintained throughout the entire replication cycle. We did not observe a trend regarding the expression level of genes belonging to large paralogous families, such as the ST kinases or Membrane Occupation and Recognition Nexus (MORN) repeat-containing proteins, a group of genes that are abundant in Marseilleviruses compared to other giant/large viruses (Boyer et al., 2009). This was because some genes have relatively high expression levels, such as MAR_ORF191, a ST-kinase, and MAR_ORF361, a MORN repeat-containing protein, the 53rd and 68th most expressed genes, respectively, while others have very low expression levels, such as MAR_ORF352, a ST-kinase, and MAR_ORF366, a MORN repeat-containing protein, the 36th and 6th lowest expressed genes, respectively.

Among the genes involved in the transcription process were transcription factors, such as transcription initiation factor IIB (TFIIB) and early transcription factor (eTF), viral RNA polymerase, and enzymes involved in the processing of RNA, such as RNA methyltransferase and mRNA capping enzyme. Some of these enzymes were expressed early, such as RNA polymerase, indicating a fast onset of viral transcript synthesis (Figure 3B and Supplementary Figure S6). Previous data have indicated that viral transcription might begin by using cell nuclear proteins, since Marseilleviruses did not carry an RNA polymerase within their virions, as reported for Noumeavirus (*Marseilleviridae*) (Fabre et al., 2017). Our data showed that genes responsible for the synthesis of the transcripts, such as RNA polymerases, were rapidly expressed, suggesting that the viral enzymes also have an important role in the transcription process, possibly acting along with and/or just after cell proteins had initiated transcription of viral genes. Curiously, most of transcription factors were expressed only after 2 h p.i. Notably, some of these factors had been detected in the MRSV particle by proteomic analysis (Boyer et al., 2009), which in association with our data suggested that these proteins might be used at the beginning of the viral replication cycle.

In contrast with other giant viruses, such as mimiviruses, klosneuviruses and tupanviruses (Raoult et al., 2004; Schulz et al., 2017; Abrahão et al., 2018), Marseillevirus have a restricted collection of genes arsenal related to the translation process, consisting of only 4 translation factors (Figure 3A). These genes were expressed from 2 h p.i., and 2 of them only after 4 h p.i., indicating their role in protein synthesis, possibly as part of the fully matured viral factory. Moreover, genes related to viral morphogenesis were also expressed at a late stage, consistent with their role in the formation of virus particles. These included those for a membrane component and the major capsid protein, one of the most expressed gene throughout the cycle (Table 1), and the core gene encoding A32-like packaging ATPase, which was involved in packaging of the viral genome into the virion (Koonin et al., 1993; Chelikani et al., 2014; Figure 3B and Supplementary Figure S6). Finally, with respect to genes involved in metabolic functions, such as those encoding lipases, proteases and proteins involved in redox reactions, the majority (16/23 = 69.5%) had a late expression pattern. For example, this was the case for class 3 lipases, peptidoglycan peptidase and thioredoxin, indicating their role in the final steps of the replicative cycle, probably in regulation of host responses to viral infection and production of new progeny.

### AT-Rich Promoter Motifs Were Not Associated With a Specific Class of Genes

**FIGURE 4:**
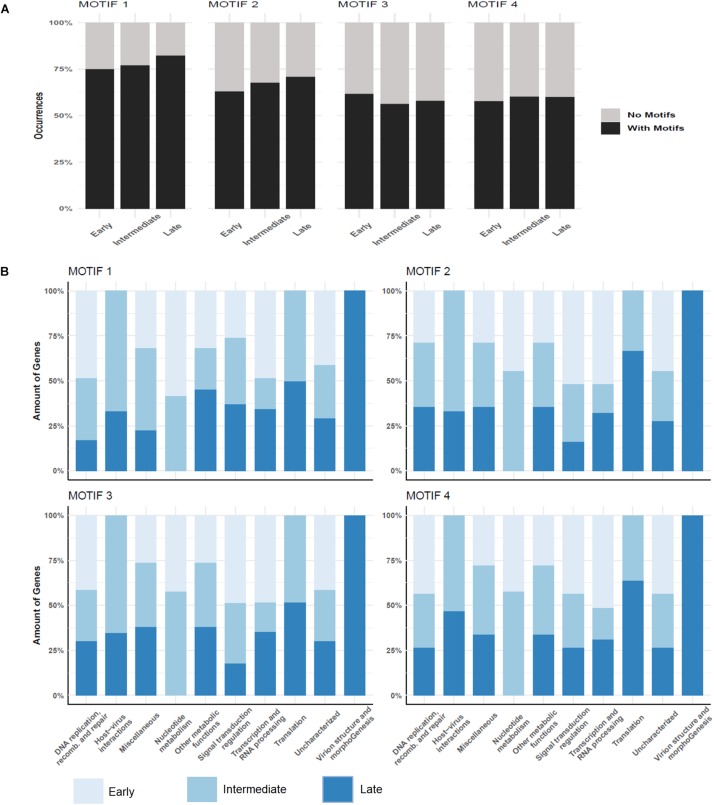
Promoter motifs association to temporal and functional classes of genes. **(A)** Distribution of four different predicted motifs according to genes belonging to different temporal categories; **(B)** Association of promoter motifs to genes belonging to different functional categories. Only genes followed by intergenic regions containing the motifs are represented. Color legend for each figure is provided in the image.

Over a half of the predicted MRSV genes were associated with an AT-rich promoter motif, AAATATTT, which had been shown to have an impact on gene expression (Oliveira et al., 2017b). At the time the motif was described, there was no information on temporal expression profiles as shown in the current study; therefore, no attempt could be made to associate the promoter to any specific class of genes, as was observed for other Megavirales representatives, such as mimiviruses and poxviruses (Davison and Moss, 1989; Knutson et al., 2006; Legendre et al., 2010). The genes that were associated to the promoter were evenly distributed around the genome, despite temporal class of genes (Figure 2). However, the promoter motif did not seem to be associated to any specific temporal class of genes since no differential distribution was observed (Figure 4A). Similarly, no specific association was found between the promoter motif and different functional categories of genes (Figure 4B). In all categories, genes were found that were not preceded by this promoter motif, in the range of 37.5–77.8%. The nucleotide metabolism and virus-host interaction categories had the lowest and highest numbers of genes associated with the promoter motif, respectively (Figure 4B). Among the 20 most expressed genes, representing about 5% of all MRSV genes, half of them are preceded by the promoter motif and the majority (15 genes) is expressed late during the replication cycle (Table 1). We evaluated the presence of the motifs of the 20 most expressed and less expressed genes and found no evident correlation between the presence of promoter motif and level of expression, i.e., there are highly expressed genes associated to the motif (10/20), as well as genes that exhibit very low expression (13/20). Therefore, although the promoter motif had been shown to be important in gene regulation (Oliveira et al., 2017b), it was not associated with any specific class of MRSV genes.

In the attempt to explain the regulation of the temporal pattern of expression observed in this study, we performed a *de novo* search for new promoter motifs in the intergenic regions of MRSV genome. We used MEME for *de novo* motif discovery to evaluate whether the previously predicted promoter motif was more represented in the intergenic regions of MRSV than what is expected by chance. MEME ranks the putative motifs detected based on both its conservation and the number of motif occurrences. MEME detected a total of four motifs with e-value smaller than cutoff (1e-5). The most significant motif MEME detected was the previously predicted promoter AAAATATTTT (1224 motif occurrences, e-value = 2.0e-170), followed by the motifs SAAARRRAAR (1484 motif occurrences, e-value = 6.3e-130), TYTYTCTTTB (1506 motif occurrences, e-value 5.7e-76) and CSAVARAV (1306 motif occurrences, e-value 1.3e-48) (Supplementary Figure S7). The promoter motifs were found in multiple copies occurring throughout the entire intergenic region, although most of them occur up to 500 nt distant from the start codon (Supplementary Figure S8). Motifs search using BaMMmotif server resulted in very similar promoter motifs as obtained using MEME software, thus corroborating the initial results (Supplementary Figure S9). Similar results were observed when we performed additional searches expanding the length of the motifs (ranging from 5 to 15 nucleotides). The core of each predicted motif was always found. We decide to use the search length range from 8 to 10 nucleotides in further analysis because it has better values among all tries. The major difference between them was the e-value for each region on each search (Supplementary Table S6). This difference was expected due to the conservative characteristics of the motif sequences. In our new analysis, the previously identified motif is associated to a larger fraction of genes, about 75% of the genes (Figure 4A). Similar to observed for the first motif we described (Oliveira et al., 2017b), the new predicted motifs are commonly found in multiple copies in different intergenic regions, which could contribute to fixing newly acquired genes, as previously hypothesized. The distribution of the new motifs regarding the temporal classes of genes was very similar to the distribution pattern of the first motif, that is, there was no significant correlation between an individual motif to any particular temporal class of genes (Figures 4A,B).

In light of the new promoter motifs and in the absence of a correlation between any of them to the temporal pattern of gene expression, we wondered if the combination of different motifs could be the temporal regulating factor. Therefore, we performed a combinatory analysis of the motifs to associate with different temporal classes of genes. However, despite the combination we searched (e.g., motif1 + motif2 or motif2 + motif4), the observed pattern was the same, with no correlation between the presence of these motifs and the temporal classes of genes (Supplementary Table S7). These results indicate that a more fine-tuning transcriptional regulation machinery might exist in the *Marseilleviridae* family.

### Marseillevirus Rapidly Alters the Transcriptional Activity of the Amoeba

**FIGURE 5:**
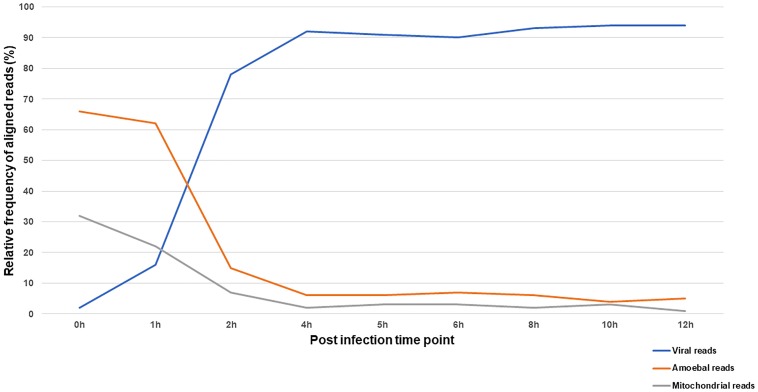
Distribution of RNA-seq reads according to their origin across the infection cycle. The relative frequency of reads aligning to Marseillevirus genome and *A. castellanii* strain Neff nuclear and mitochondrial genomes during the infection cycle.

The reaction of the amoeba to infection by Marseillevirus was observed through the compared transcriptional activity of the cells and the virus during the course of infection. The transcriptional activity was inferred from the proportion of reads that could be assigned to either the amoeba or the virus. In total, 35 million of reads generated from ribodepleted RNA extract of cells collected at 9 different times of the viral cycle were analyzed. Reads were sequentially mapped against the viral genome then *Acanthamoeba castellanii* strain Neff genomic and mitochondrial sequences. The relative frequencies of confidently aligned reads are represented in Figure 5 and the absolute number of reads and their distribution by sample to their assigned origin is presented in Supplementary Table S8.

At 0 h p.i. (30 min after virus adsorption), over 95% of aligned reads were cellular, of genomic or mitochondrial origin. This shows an active transcriptional activity of the amoeba during virus entry paralleled with a minimal yet detectable viral transcription. The first hour of infection was marked by a gradual increase in transcripts of viral origin and a decrease in cellular transcripts. The alteration of cellular transcriptional activity was sharp at 2 h p.i. and leveled off at 4 h p.i. with viral reads representing more than 90% of the aligned reads. Interestingly, mitochondrial and genomic transcripts of the amoeba followed the same kinetics suggesting a reduction in both cellular transcription and energy metabolism during infection by Marseillevirus.

### Structural Genes of the Amoebal Translation Apparatus Are Downregulated

**FIGURE 6:**
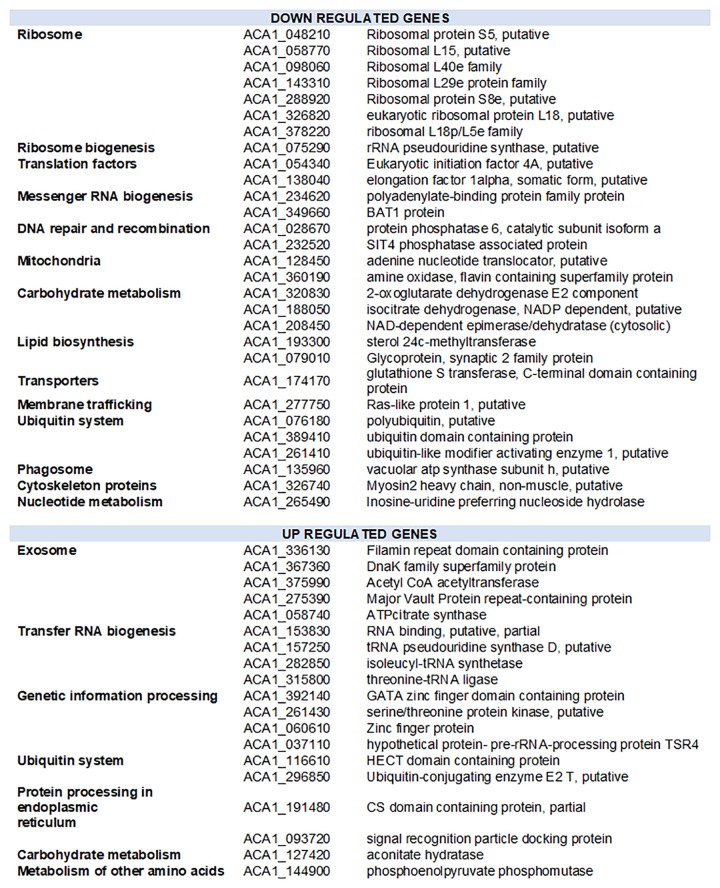
Functional distribution of amoebal genes with altered expression during the infection by Marseillevirus. Downregulated and upregulated genes correspond to genes with expression reduced or increased by at least 1 log2-fold-change and False Discovery Rate of 0.05 in the pairwise comparison of grouped samples from early, intermediate and late infection time points.

To further investigate the amoebal response to infection, we analyzed the evolution of the transcriptional activity at the gene level using he reference annotation of Acanthamoeba castellanii Neff genome that includes 14973 genes. Only 3036 genes could be assigned at least one read in at least one dataset. The expression data for amoebal genes are depicted in Supplementary Table S9. Three groups of samples could be defined regarding the amoebal gene expression using an unsupervised clustering of samples: early group (0, 1, and 2 h p.i.), intermediate group (4, 5, 6, and 8 h p.i.) and late group (10 and 12 h p.i.). In absence of biological replicates and considering the intragroup similarities observed (average biological coefficient of variation of 0.3), the differential gene expression analysis was performed between the three temporal groups. The Multidimensional scaling plot that allowed the identification of the three temporal groups is presented in Supplementary Figure S10. A total of 48 amoebal genes showed significant change in their expression between the three temporal groups (Figure 6 and Supplementary Figure S11). Among these differentially expressed genes, 28 showed an overall decrease in their expression levels. The genes identified as down regulated encode for proteins linked to the translation apparatus of the cell, including seven structural constituents of the ribosome such as ribosomal proteins S8e and L15, proteins that bind to the rRNA within the ribosomes including S5 and 18p/L5c and the rRNA pseudouridine synthase that plays a major role in the biogenesis of the ribosome (Anderson et al., 2005).

*Acanthamoeba castellanii* Neff strain genome is predicted to harbor four paralogs of the eukaryotic initiation factor 4A, a translation factor that binds, within the initiation complex, to the 5′ cap of the mRNA and recruits it for its translation by the ribosome (Gingras et al., 1999). One of these paralogs (ACA1_054340) appeared downregulated during the infection of the amoeba by MRSV along with other proteins responsible of the mRNA export out of the nucleus, such as the polyadenylate-binding protein (ACA1_234620) and the BAT1 protein (ACA1_349660) which, besides, regulates transcription elongation and mRNA splicing. Parallel to a reduction in carbohydrate metabolism and lipid biosynthesis related proteins, nuclear genes encoding for proteins involved in mitochondrial energy transfer pathways were downregulated. Another notable gene with reduced transcription upon MRSV infection is the Ras like protein 1 (ACA1_277750), a protein predicted to act on membrane trafficking and micropinocytosis in *Dictyostelium discoideum* (Williams et al., 2019). This gene has a significant decrease in expression in the late phase of infection after a plateau in early and intermediate times. The late downregulation of this gene could be related to the virus manipulation of the amoebal reaction in the early steps of infection.

On the other hand, 19 genes of the amoeba showed a significant increase in expression during the infection cycle. Genes predicted as upregulated were of different functional groups, including transfer RNA biogenesis related proteins such as isoleucyl-tRNA synthase (ACA1_282850) and threonine tRNA ligase (ACA1_315800) (Figure 6). Interestingly, five proteins linked to exosome secretion showed an increase in expression during the infection with MRSV. In *Acanthamoeba castellanii*, exosome secretion has been reported in nutritional stress and was associated to the amoebal adaptation to hostile environment (de Gonçalves et al., 2018). Supplementary Figure S12 shows the evolution of the expression of the 48 genes identified as differentially expressed between the three temporal groups.

### Mitochondrial Expression Is Time-Independent at the Gene Level

To identify a pattern of expression for mitochondrial genes of the amoeba during Marseillevirus infection, the normalized counts of the 53 described genes were generated. Expression levels of the mitochondrial genes varied largely across samples (min = 17; max = 220,489; median = 5,136). The inspection of pattern of expression for these genes revealed an absence of correlation with the time of infection. Although the overall mitochondrial gene expression is decreased during the infection by Marseillevirus, the proportionality of expression between 3 groups of genes appears maintained (Supplementary Figure S12). This group of genes defines three levels of expression: (i) Genes with high expression level (*n* = 22) with a median TPM count of 31,364; (ii) Genes with medium expression level (*n* = 19) with a median TPM of 2,719; and (iii) Genes with low expression level (*n* = 12) with a null median TPM value. It is noteworthy that all genes belonging to the third group are tRNA encoding genes. The low detection of corresponding reads could therefore be due to their secondary structure. Genes of the first group correspond to energy metabolism or ribosomal RNA. Most ribosomal proteins encoded by the mitochondrial genome belonged to the second group of genes. The complete normalized read counts data and functional distribution of genes are presented in Supplementary Table S10.

## Discussion

To elucidate the transcriptional landscape of Marseilleviruses, we initially employed the RNA-seq approach, a technique that has been widely used, and has undergone almost constant improvement in this age of high-throughput sequencing (Conesa et al., 2016; Todd et al., 2016). To avoid a massive sequencing of ribosomal RNA (rRNA), a major component of the cellular RNA pool, we used the rRNA depletion strategy, which has never previously been used in studies involving giant viruses and amoebae. The method proved to be efficient and allowed important features of Marseillevirus transcription to be revealed. In view of the high coverage of RNA sequence reads mapped to the viral genome in this work, it is possible that the intergenic regions are shorter than anticipated, as observed in phycodnaviruses, another member group of the NCLDVs (Blanc et al., 2014). The temporal expression profile observed for Marseillevirus T19, with genes divided into early, intermediate and late categories, was similar to that observed for other giant DNA viruses (Oliveira et al., 2017a). This profile was validated by RT-qPCR data, a more sensitive technique compared to high-throughput sequencing. Some genes are likely to be transcribed in different moments during the course of infection, e.g., early and intermediate moments or intermediate and late periods. This multistage expression pattern was observed in our RT-qPCR validation assay, wherein the tested genes have a significant expression level in a given moment despite the expression peak is well defined. It is noteworthy that this three-wave of transcription appears to be a common feature among large and giant DNA viruses, despite differences in the replication cycle especially the location of viral factories establishment, and new studies involving recent isolated viruses could bring more exciting novelties to the field.

Our data confirmed the existence of all 457 genes initially predicted for MRSV, with a minimum of 10 sequence reads for each putative gene. It also showed an incredibly rapid onset of gene expression, with all genes being expressed within 4 h p.i. Other NCLDVs also exhibit a fast transcriptional activity, with many genes being expressed as soon as 30 min post-infection, as observed for some algae viruses from *Phycodnaviridae* and *Mimiviridae* families, and also poxviruses (Assarsson et al., 2008; Blanc et al., 2014; Moniruzzaman et al., 2018). It is interesting to note that not every NCLDVs exhibit that fast onset on gene expression, as observed for Ostreococcus tauri virus 5 (*Phycodnaviridae*), which have two phases of transcription, the majority of genes being expressed after 9 h our infection (Derelle et al., 2018). Considering that distinct cellular structures involved in virus production, previously termed virus factories, are observed for these viruses around 4 h p.i. (Arantes et al., 2016; Fabre et al., 2017), many of the viral genes are probably being expressed outside a location where virus is being actively produced, presumably in the host cytoplasm. It is possible that a fraction of early transcripts is carried within the virus particles to the host cytoplasm. Preliminary data identified only a few transcripts inside marseillevirus capsid (Boyer et al., 2009), but a more in-depth analysis must be done to better investigate this aspect. Interestingly, the late viral genes were expressed from 4 h p.i, but remained active for at least 12 h p.i. It was possible that the maintenance of the expression of these genes was performed inside the viral factory, where the mRNAs would be less subjected to degradation by the cellular machinery. Genes belonging to different functional classes had peaks of expression at varying times, many of them exhibiting an expected pattern, such as translation and viral morphogenesis genes that were mostly expressed late in infection. However, some classes had genes that were expressed at different times, such as those involved in DNA replication, repair, and recombination processes. This profile showed that these genes were important throughout the entire viral cycle, some being used at the beginning of the cycle and others being used later, whose products could be packaged into the viral particle, as already verified for the viral DNA polymerase (Boyer et al., 2009). In addition, we observed that MRSV genes were not organized in the genome according to their functions, or regarding their temporal expression profiles. This reinforced the idea of intra-genomic mosaicism previously proposed for these viruses (Boyer et al., 2009).

Notably, the majority of MRSV genes with highest level of expression had no known function, representing hypothetical proteins. Curiously, two of the most expressed genes with predicted function have zinc-finger domains. Zinc-related proteins have different functions, including regulation of transcriptional activity (Laity et al., 2001). Both zinc-finger proteins from MRSV are expressed in late moments of the replication cycle, suggesting that their products can be involved in a fine-tuning regulation of other genes’ transcription. Overall, a substantial part of the most abundant part of the marseillevirus transcriptome represents a “dark transcriptome.” It indicates that the most abundant part of the MRSV transcripts encode for putative proteins that remain to be structurally and functionally studied. A similar pattern has also been observed in all transcript profiling studies performed to date for giant viruses, such as mimiviruses, mollivirus and pithoviruses (Legendre et al., 2010, 2014, 2015). This showed that a substantial part of the gene content of these viruses differed considerably from that of other similarly characterized living organisms, thus representing new expressed elements. Our data clearly demonstrate that MRSV ORFan genes are *bona fide* expressed, thus not considering junk DNA neither bioinformatic errors in initial gene prediction step.

Similar to other NCLDVs, Marseilleviruses have a well-conserved AT-rich promoter motif, which is associated with over 50% of the genes (Oliveira et al., 2017b). Our new data show that this promoter is even more abundant than previously predicted, leading us to consider it as a core motif. It is worthy to note that this motif is very similar to the palindromic hairpin sequence identified in noumeavirus and melbournevirus genome, acting as a polyadenylation signal (Fabre et al., 2017). The promoter activity of this core motif was experimentally demonstrated in a previous work, thus making its participation as a termination signal very unlike (Oliveira et al., 2017b). Despite our efforts, we have not found any associated with any specific temporal class of genes. This lack of association contrasted with what was observed for another amoeba giant virus (mimivirus), in which very conserved AT-rich promoter motifs was mostly associated with early genes (Legendre et al., 2010). We found other promoter motifs by using a *de novo* search strategy, but none of these motifs was significantly associated to any temporal class of genes. The presence of AT-rich promoters in a genome with a higher GC content (45%) than for mimivirus genomes, and its non-correlation with any temporal class, suggested the existence of other regulatory mechanisms yet to be discovered for Marseillevirus. Different mechanisms might be involved and deserve further investigation, including the presence of additional upstream regulatory elements such as enhancers; differential expression and/or usage of transcription factors; and also the possible involvement of additional coactivators/correpressors of transcription specific for each moment of the infectious cycle.

During the infection cycle, Marseillevirus seems to alter its host transcription, translation and energy metabolism with a global decrease in expression of amoebal nuclear and mitochondrial genes. To overcome the limit of use of a single dataset per infection time, we used the temporal clustering of samples to group infection times sharing similar expression pattern of genes. The limit of this approach is a higher dispersion than that expected of exact biological replicates which reduces the identification of differentially expressed genes. Nevertheless, it offers a first insight into modification of host expression. Indeed, using this approach on *A. castellanii* nuclear genes expressionwe identified a clear transcription downregulation of some ribosomal protein encoding genes similar to what is reported in amoeba undergoing encystment (Schulze and Jantzen, 1982; Matthews et al., 1995). Parallelly, the mitochondrial transcription showed a global decreased, while maintaining the general distribution of gene expression levels between functional groups of genes independently of the infection time. The transcription of genes linked to exosome secretion is increased suggesting that an infected amoeba sends danger signals to other amoebae to reduce the propagation of the virus.

These observations suggest that MRSV developed a rapid expression of its transcription and translation factors as an adaptation system to escape the amoebal defense mechanism. This early expression could subsequently silence the transcription of the host without affecting the viral cycle. The reduction of transcription could otherwise be a defense mechanism of the amoeba. It is also appears that the virus takes control of the amoebal tRNA biogenesis pathway, by increasing the transcription of corresponding genes early during infection. This allows the virus to have a productive replication cycle despite the amoebal response.

Altogether this study provided new information about the biology of a newly discovered group of viruses, suggesting the existence of a much more complex transcriptional machinery than originally envisaged, and a complex process of virus-host interaction at molecular level. Advances in RNA sequencing and *in silico* analysis technologies and analytical tools may provide important insights into the molecular mechanisms exhibited by Marseilleviruses, especially in the context of interactions with their amoeba host and other sympatric organisms.

## Data Availability Statement

The sequencing data from this study have been submitted to the NCBI Short Read Archive (http://www.ncbi.nlm.nih.gov/Traces/sra/sra.cgi) under the accession number ERP117375.

## Author Contributions

RR and AL performed the biological experiments. RR, AL, AP, and GO performed the bioinformatics analyses. FL and PC supervised the bioinformatics analyses. BL and JA conceived and supervised the project. RR and AL wrote the manuscript. BL, PC, and JA revised the manuscript. All authors read and approved the final version of the manuscript.

## Conflict of Interest

RR, PC, BS, and JA are members of a CAPES-COFECUB project. JA was a CNPq researcher. The remaining authors declare that the research was conducted in the absence of any commercial or financial relationships that could be construed as a potential conflict of interest.

## Footnotes

1 https://www.ncbi.nlm.nih.gov/tools/primer-blast/

## Supplementary Material

FIGURE S1 One-step growth curve of Marseillevirus. **(A)** Genome replication represented by ΔCt values; **(B)** Viral replication represented by viral titer values expressed at TCID_50_/mL.

FIGURE S2 Ribosomal depletion analysis by Agilent bioanalyzer. **(A)** Different kits for ribosomal depletion were tested for *Acanthamoeba castellanii* cells, and the bacteria kit exhibited the best results, thus the chosen one for further use; **(B)** Digital electrophoresis of RNA treated with Ribozero bacteria kit for each time used for RNA-seq analysis, evidencing the absence of ribosome in all samples prior sequencing.

FIGURE S3 Protocol with full parameters used at HISAT2 for RNA-seq reads alignment.

FIGURE S4 Number of genes vs. number of total corresponding reads (cumulative). Cumulative distribution of total corresponding reads to the genes of Marseillevirus. Before normalization the number of reads per gene ranged from 15 to 372,331.

FIGURE S5 Validation of RNA-seq data by RT-qPCR assays. Molecular assays were performed to validate the temporal profile for gene expression of Marseillevirus using different genes. Gray boxes highlight the peak of activation of the genes, indicating the **(A,B)** “Early” genes; **(C,D)** “Intermediate” genes; and **(E,F)** “Late” genes. The expression levels are depicted as arbitrary units, calculated using the ΔCt method. Time 0 h corresponds to 30 min of infection due to virus adsorption period. All assays were performed twice. Error bars indicate standard deviation.

FIGURE S6 Expression level of different genes based on RNA-seq data. The expression level is based on TPM values of each gene considering different times of infection (0 h corresponds to 30 min of infection due to virus adsorption period). The time where the expression level was the highest was considered 100% and the other values were relative to this one. **(A)** D6/D11 helicase; **(B)** DNA polymerase B family; **(C)** DNA topoisomerase II; **(D)** Alkylated DNA repair protein; **(E)** RNA polymerase α subunit; **(F)** RNA polymerase β subunit; **(G)** Transcription Factor IIB; **(H)** eukaryotic Transcription Factor; **(I)** Ribonucleotide reductase small chain; **(J)** Nucleotide deaminase; **(K)** Major Capsid Protein; **(L)** A32-like packaging ATPase; **(M)** eukaryotic Initiation Factor 5; **(N)** eukaryotic Release Factor 1; **(O)** Elongation factor 1α. a.u.: arbitrary units.

FIGURE S7 Promoter motifs found in Marseillevirus genome using MEME software. The motifs are presented from the most representative to the less, considering the e-value cutoff of 1e-5 established during the *de novo* search using MEME suit. **(A)** motif 1; **(B)** motif 2; **(C)** motif 3; **(D)** motif 4.

FIGURE S8 Position of predicted promoter motif 1 **(A,B)**; motif 2 **(C,D)**; motif 3 **(E,F)**; and motif 4 **(G,H)**. Histograms evidencing the location of motifs in multiple copies considering all intergenic regions are depicted in panel **A,C,E,G**. Histograms evidencing the location of motifs up to 500 nucleotides from the start codon are depicted in panels **B,D,F,H**. Motifs are separated by the temporal class of the respective genes.

FIGURE S9 Promoter motifs found in Marseillevirus genome using BaMMmotif server. The motifs are presented from the most to the less representative. AvRec (Average Recall) - To avoid false predictions, the BaMM software determines true and false positive ratios (sensitivity and specificity) for each predicted promoter, a mean model recall averaged over range precision from 0 to 1 (*R* = TP/FP); estimates the false discovery rate (FDR) and then compute the log10R-values as a quality measure. Frac. occurrence (Fraction of sequences with motif) - Shows the occurrence (from 0 to 1) of the motif in the analyzed sequences.

FIGURE S10 Multi-dimensional scaling of *Acanthamoeba castellanii* Neff RNA-seq read counts showing the three temporal groups: - early group in black with 0, 1, and 2 h infection time (0 h corresponds to 30 min of infection due to virus adsorption period); intermediate group (red) with 4, 5, 6, and 8 h infection time; and late group (green) 10 and 12 h infection time.

FIGURE S11 Expression levels of up and down regulated genes of Acanthamoeba castellanii during the course of infection by Marseillevirus. The function of each gene is depicted in Table 1.

FIGURE S12 Acanthamoeba castellanii neff mitochondrion gene expression classes. Heatmap of mitochondrial gene expression profiles. Rows correspond to the 53 mitochondrial genes and columns to the 9 infection time points (0 h corresponds to 30 min of infection due to virus adsorption period). Expression profiles are clustered using Euclidean distance and average linkage of log-transformed normalized expression counts. A dendrogram of the clustering is shown on the left. Genes could be partitioned into three main group, “Genes with high expression levels” (top), “Genes with intermediate expression level” (center), and “Genes with low expression levels” (bottom). Expression levels are displayed from Red (low expression) to yellow (high expression). The function of each gene related to their level of expression pattern can be found in Supplementary Data Sheet S1.

TABLE S1 Genes and primers used in RT-qPCR reactions.

TABLE S2 Expression data in TPM for all genes at each evaluated time of infection (0 h corresponds to 30 min of infection due to virus adsorption period).

TABLE S3 The top 20 most highly expressed annotated Marseillevirus genes. The expression data are represented as fractions Top 20 most expressed annotated genes. Expression level [(number of normalized reads of a time/total number of normalized reads of the gene) × 100] of each gene is exhibited. The raw data in Transcript Per Million reads are depicted in Table 1 (0 h corresponds to 30 min of infection due to virus adsorption period). N: no; Y: yes; E: early; I: intermediate; L: late.

TABLE S4 Predicted function of the 20 most expressed genes evaluated by different approaches: blastp, interproscan and delta blast. Coverage and identity values for best hits at blastp are shown.

TABLE S5 Marseillevirus genes for which a valid transcript could be assembled using the Trinity-PASA pipeline. For each gene, the genomic coordinates of the corresponding transcript are reported. The five prime and three prime Untranslated Transcribed Regions (UTRs) coordinates are listed when available.

TABLE S6 Promoter motif search using MEME software considering sequence size of 8∼10 and 5∼15. Motif sequences are ordered according to statistical e-value.

TABLE S7 Statistical analysis of promoter motifs combination in association to different temporal classes of genes.

TABLE S8 Assignment of RNA-seq reads according to their origin across samples. The absolute number of generated RNA-seq reads and reads mapping to the viral amoebal nuclear and mitochondrial genome is presented as well as the percentage of each fraction from the assigned reads.

TABLE S9 Expression data in TMM for all *Acanthamoeba castellanii Neff* genes at each evaluated time of infection (0 h corresponds to 30 min of infection due to virus adsorption period).

TABLE S10 Expression data in TPM for all *Acanthamoeba castellanii Neff* mitochondrial genes at each evaluated time of infection (0 h corresponds to 30 min of infection due to virus adsorption period). The level of expression group and functional annotation of each gene is included.

DATA SHEET S1 Gene function and temporal class (complement to Figure 2). The order of genes presented here by each temporal class is related to the order they are shown in Figure 2B.
