# Supplementary figures and images for: Analysis of a Marseillevirus Transcriptome Reveals Temporal Gene Expression Profile and Host Transcriptional Shift
Source: Front Microbiol. 2020 Apr 14;11:651. doi: 10.3389/fmicb.2020.00651 (PMC7192143; doi:10.3389/fmicb.2020.00651)

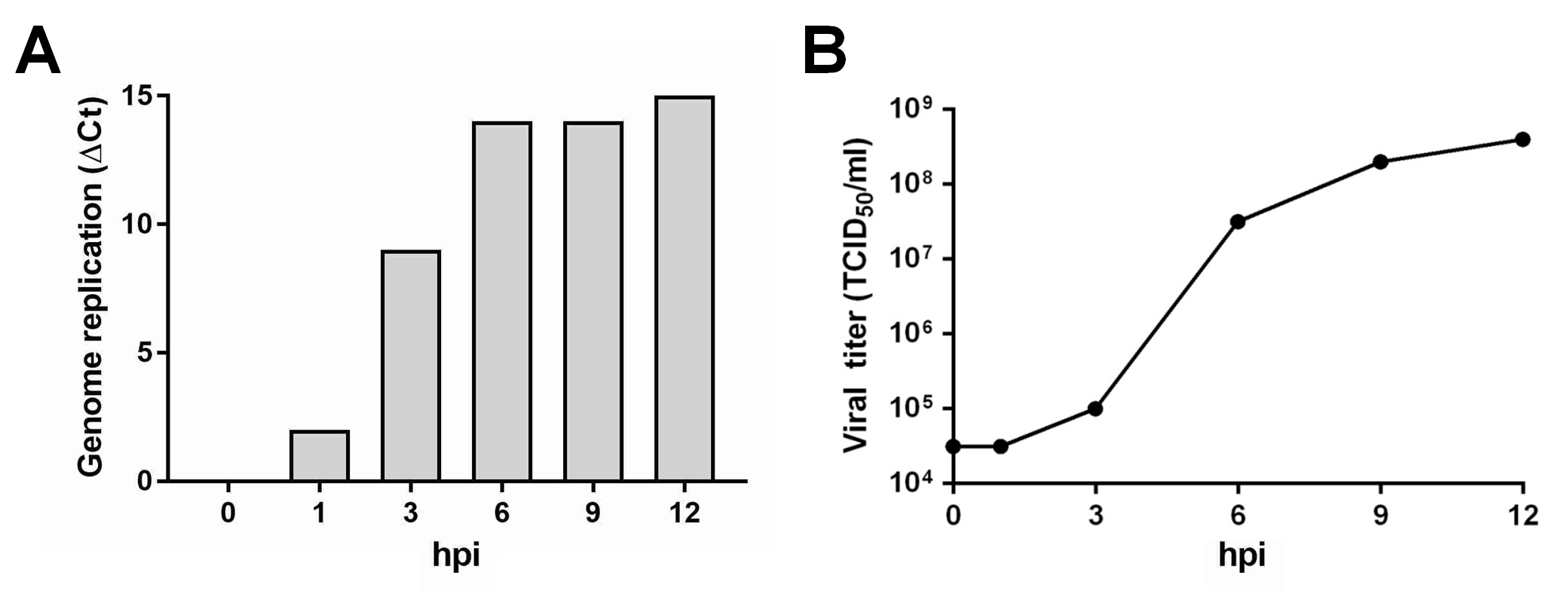

Supplement: FIGURE S1 — One-step growth curve of Marseillevirus. (A) Genome replication represented by ΔCt values; (B) Viral replication represented by viral titer values expressed at TCID50/mL. [file Image_1.TIF]

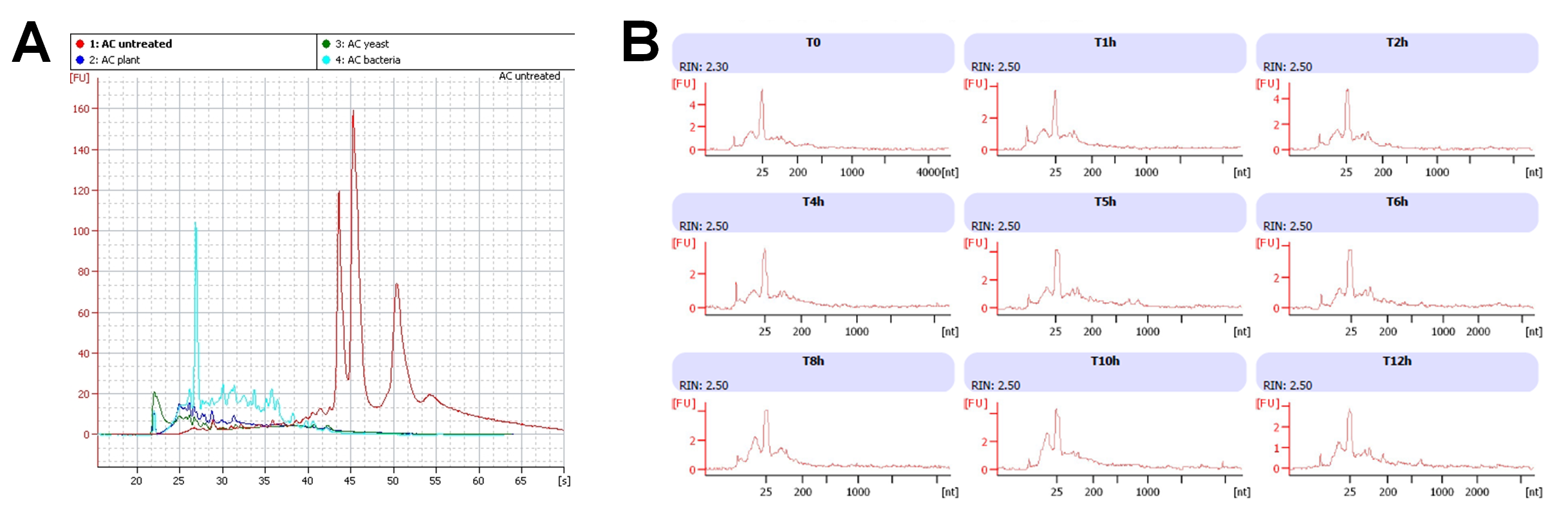

Supplement: FIGURE S2 — Ribosomal depletion analysis by Agilent bioanalyzer. (A) Different kits for ribosomal depletion were tested for Acanthamoeba castellanii cells, and the bacteria kit exhibited the best results, thus the chosen one for further use; (B) Digital electrophoresis of RNA treated with Ribozero bacteria kit for each time used for RNA-seq analysis, evidencing the absence of ribosome in all samples prior sequencing. [file Image_2.TIF]

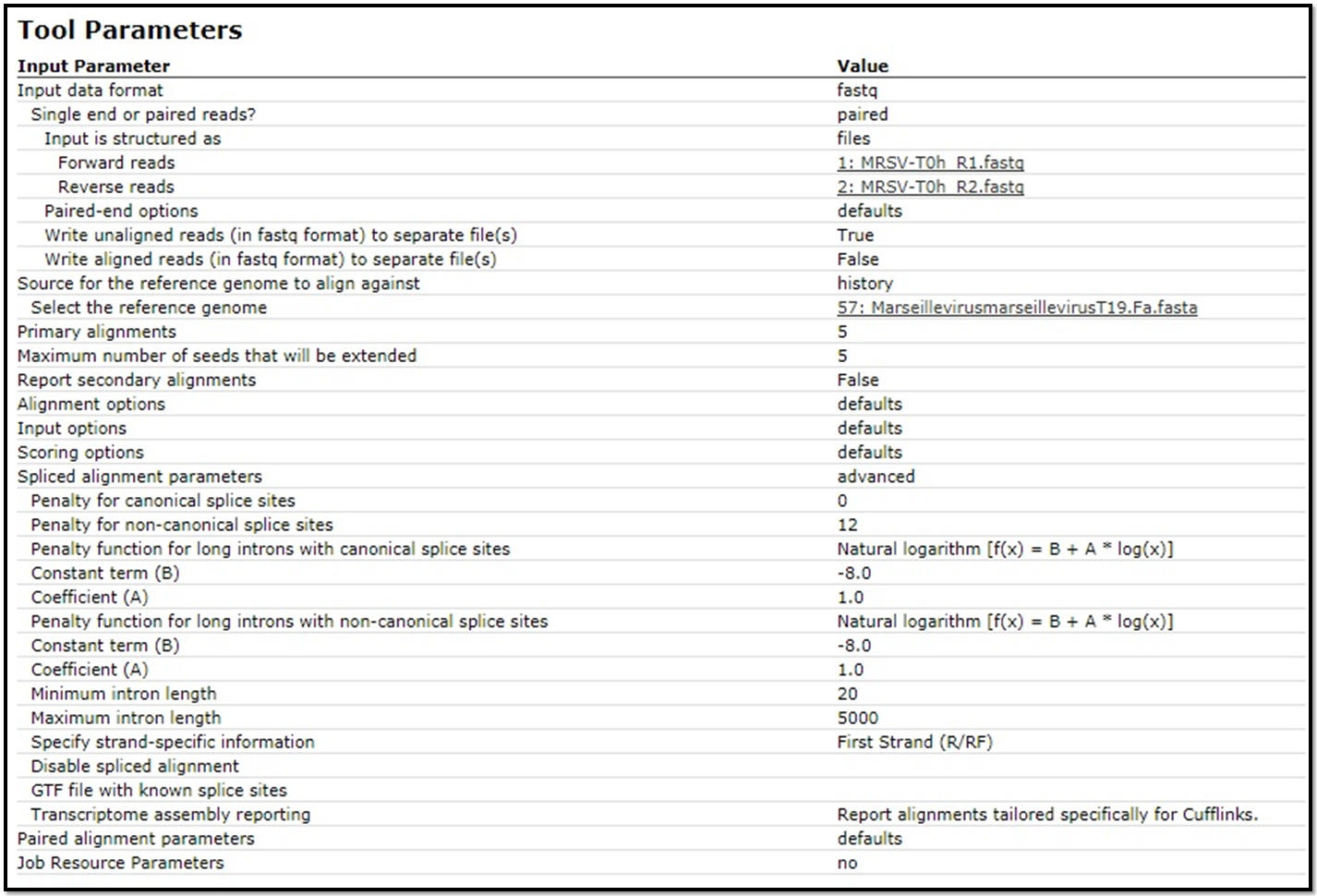

Supplement: FIGURE S3 — Protocol with full parameters used at HISAT2 for RNA-seq reads alignment. [file Image_3.TIF]

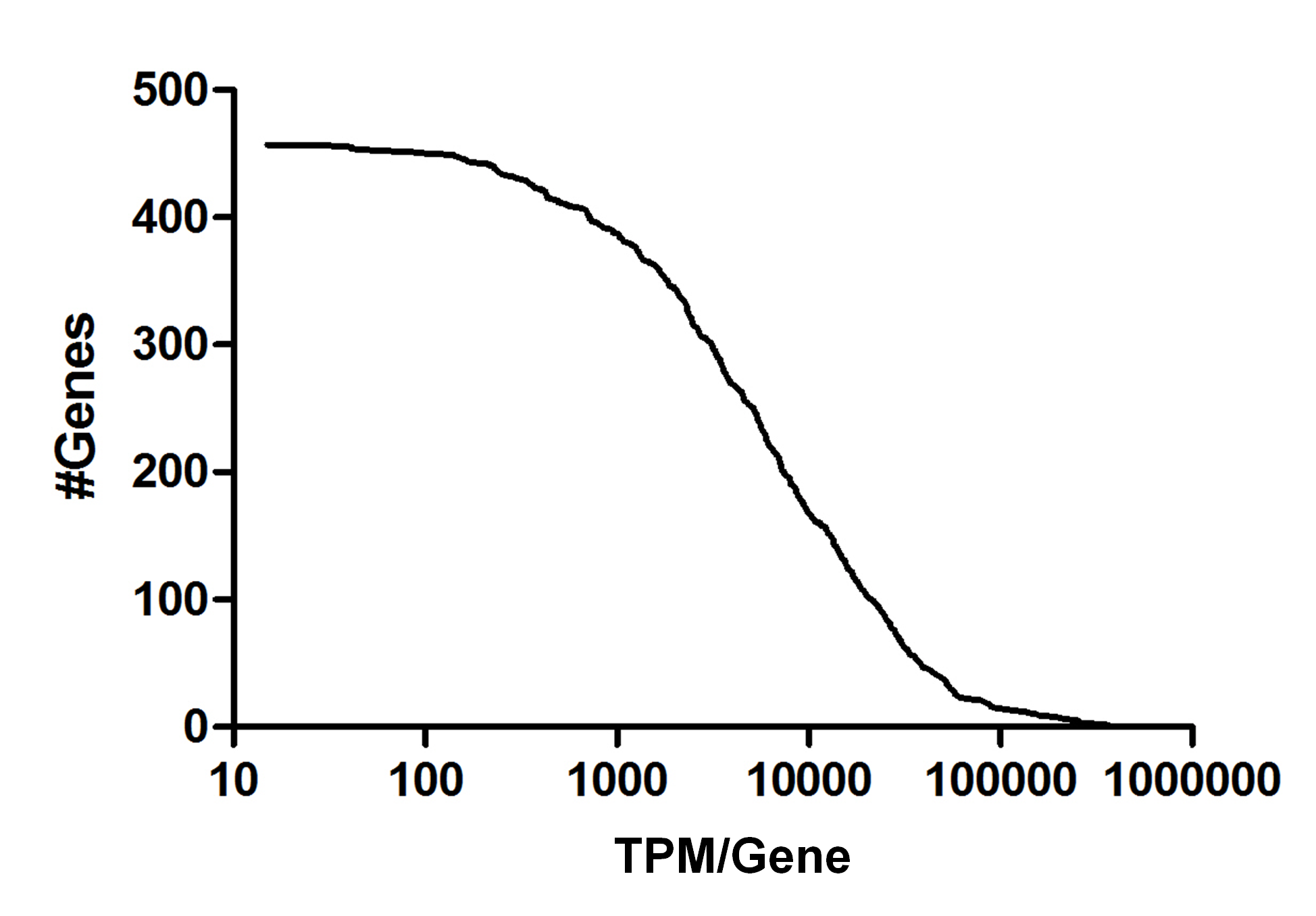

Supplement: FIGURE S4 — Number of genes vs. number of total corresponding reads (cumulative). Cumulative distribution of total corresponding reads to the genes of Marseillevirus. Before normalization the number of reads per gene ranged from 15 to 372,331. [file Image_4.TIF]

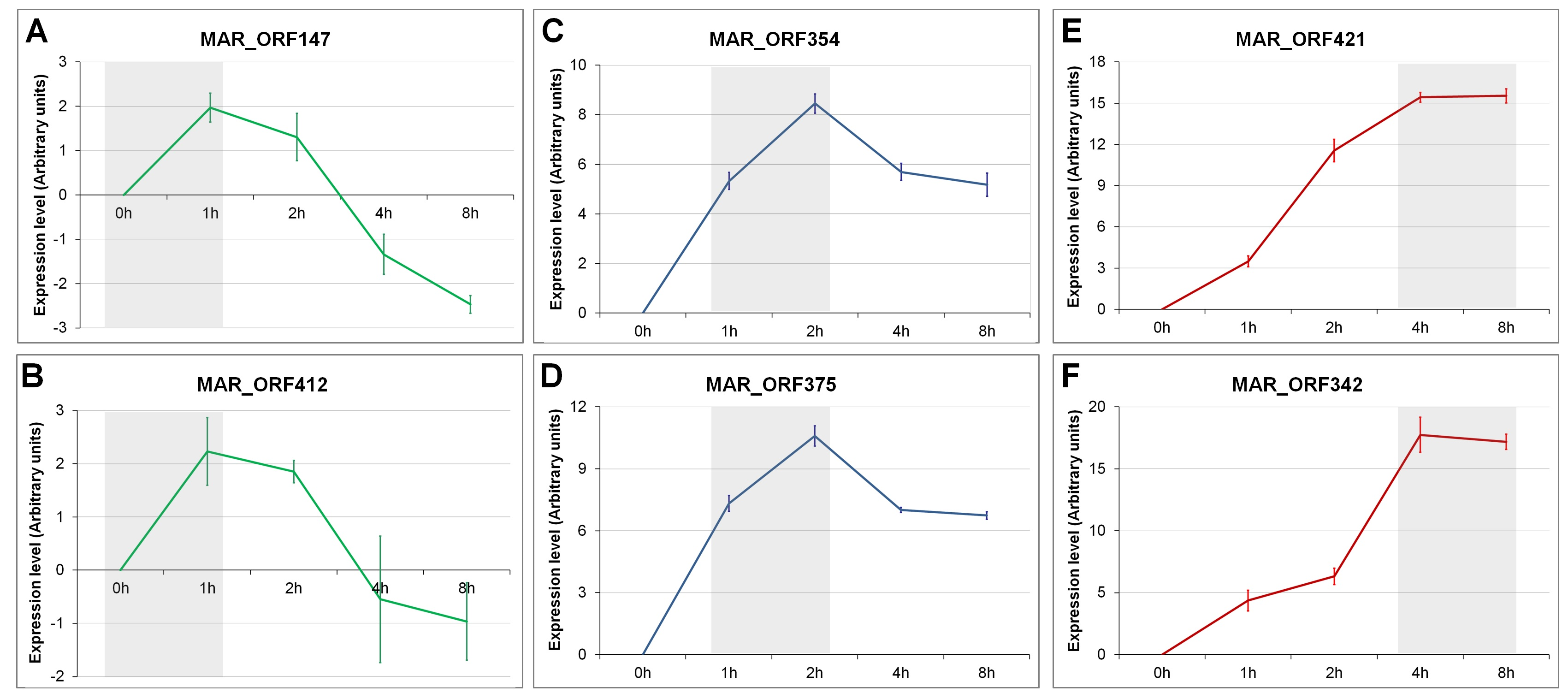

Supplement: FIGURE S5 — Validation of RNA-seq data by RT-qPCR assays. Molecular assays were performed to validate the temporal profile for gene expression of Marseillevirus using different genes. Gray boxes highlight the peak of activation of the genes, indicating the (A,B) “Early” genes; (C,D) “Intermediate” genes; and (E,F) “Late” genes. The expression levels are depicted as arbitrary units, calculated using the ΔCt method. Time 0 h corresponds to 30 min of infection due to virus adsorption period. All assays were performed twice. Error bars indicate standard deviation. [file Image_5.TIF]

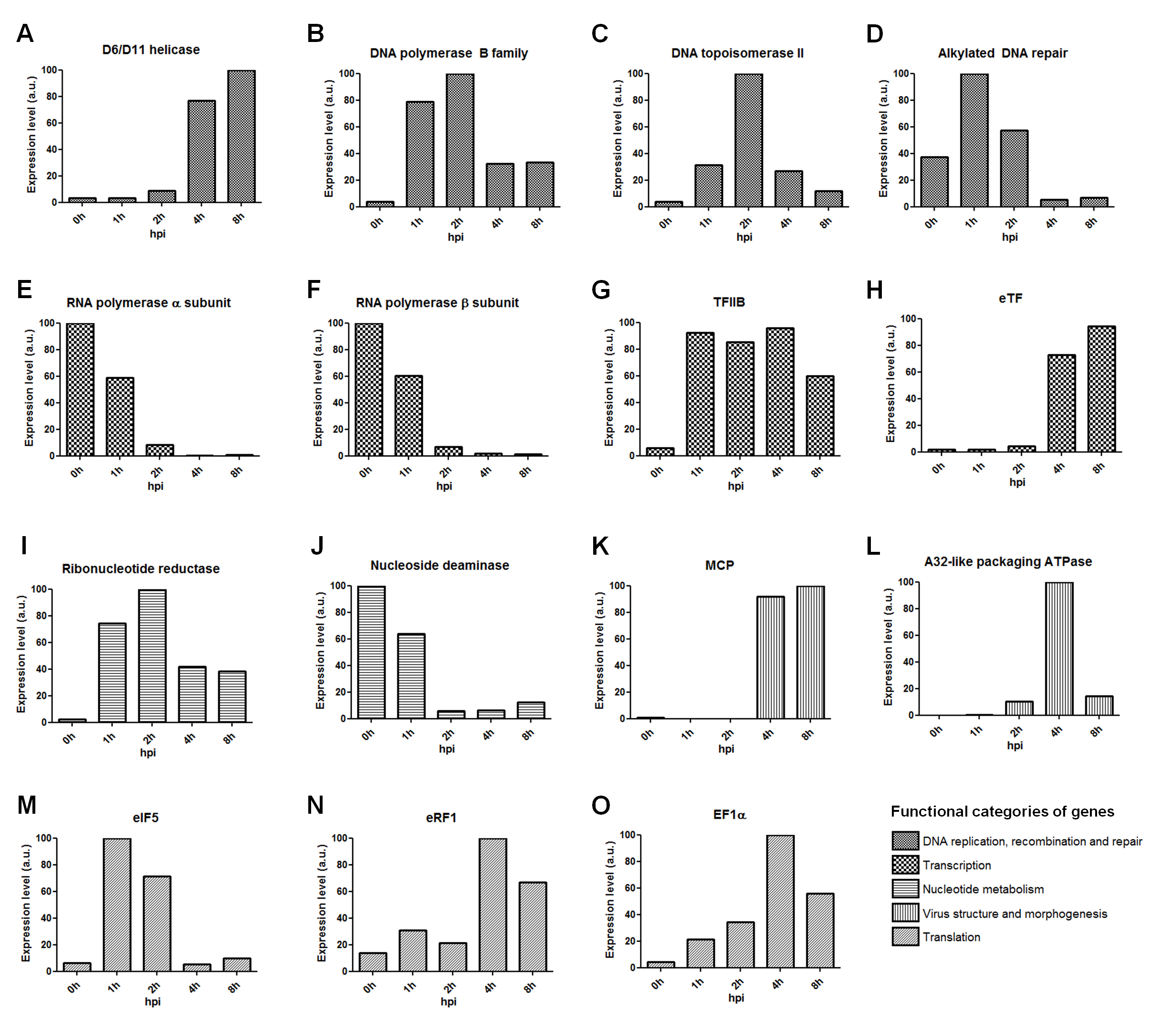

Supplement: FIGURE S6 — Expression level of different genes based on RNA-seq data. The expression level is based on TPM values of each gene considering different times of infection (0 h corresponds to 30 min of infection due to virus adsorption period). The time where the expression level was the highest was considered 100% and the other values were relative to this one. (A) D6/D11 helicase; (B) DNA polymerase B family; (C) DNA topoisomerase II; (D) Alkylated DNA repair protein; (E) RNA polymerase α subunit; (F) RNA polymerase β subunit; (G) Transcription Factor IIB; (H) eukaryotic Transcription Factor; (I) Ribonucleotide reductase small chain; (J) Nucleotide deaminase; (K) Major Capsid Protein; (L) A32-like packaging ATPase; (M) eukaryotic Initiation Factor 5; (N) eukaryotic Release Factor 1; (O) Elongation factor 1α. a.u.: arbitrary units. [file Image_6.TIF]

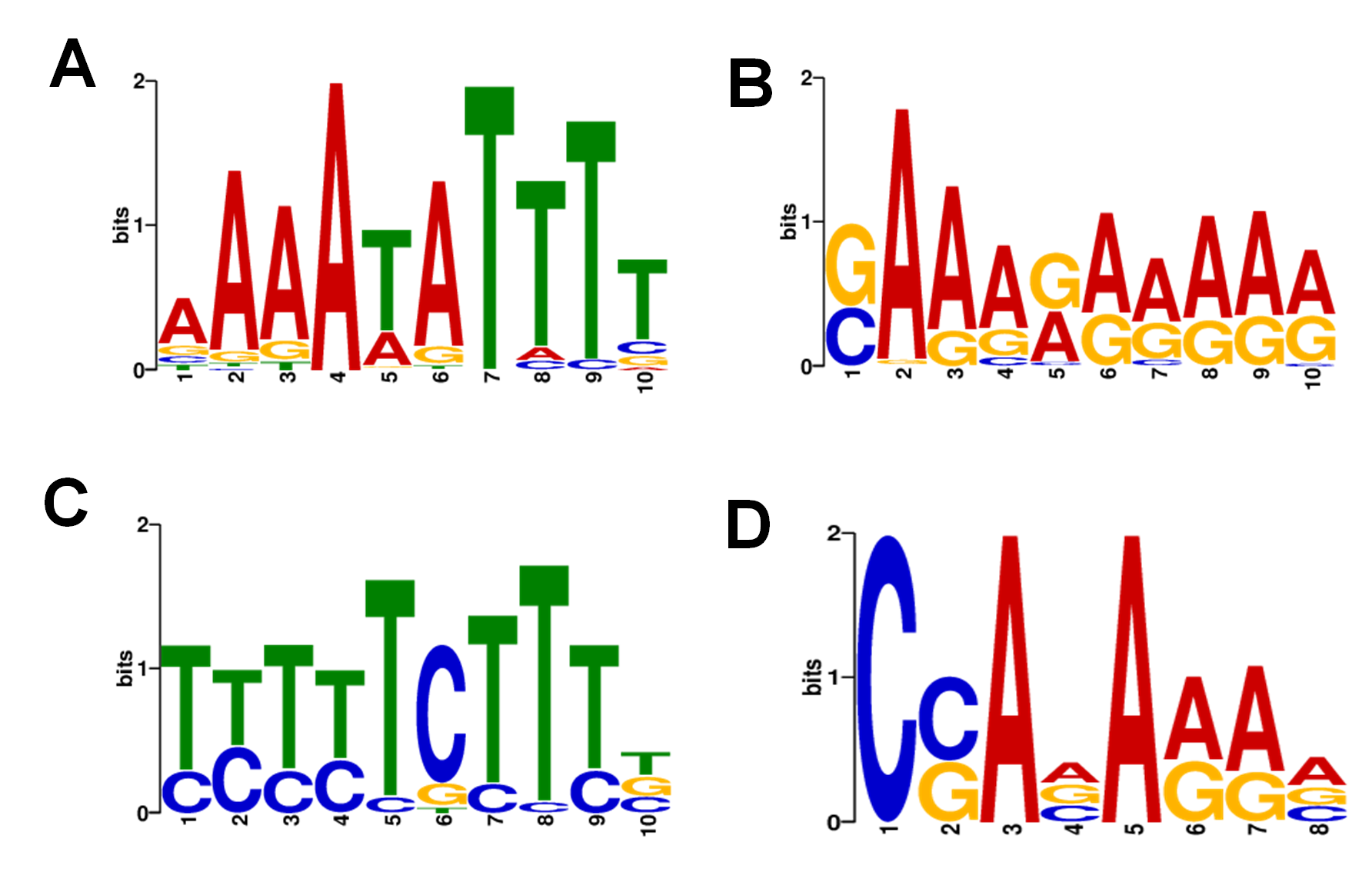

Supplement: FIGURE S7 — Promoter motifs found in Marseillevirus genome using MEME software. The motifs are presented from the most representative to the less, considering the e-value cutoff of 1e-5 established during the de novo search using MEME suit. (A) motif 1; (B) motif 2; (C) motif 3; (D) motif 4. [file Image_7.TIF]

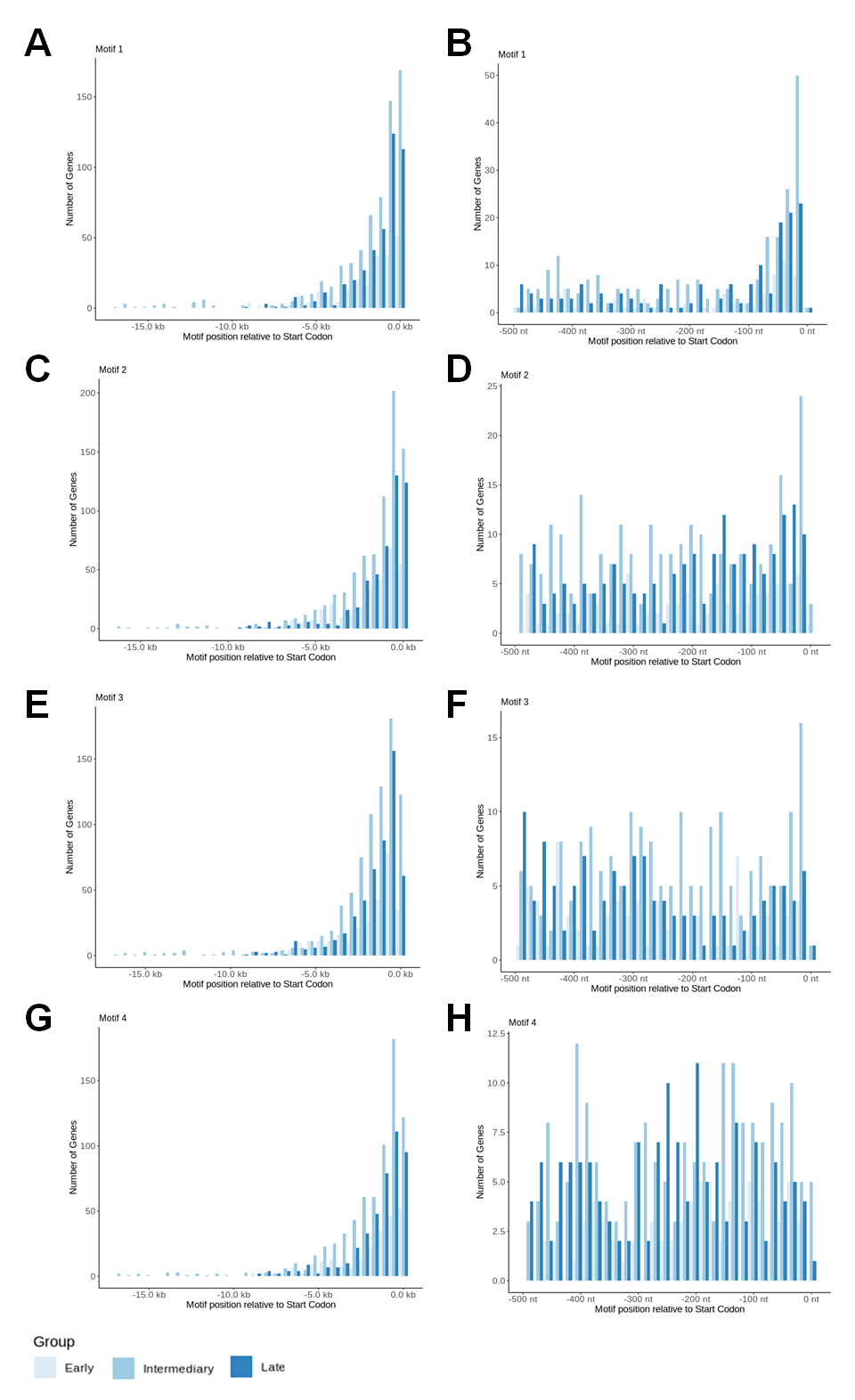

Supplement: FIGURE S8 — Position of predicted promoter motif 1 (A,B); motif 2 (C,D); motif 3 (E,F); and motif 4 (G,H). Histograms evidencing the location of motifs in multiple copies considering all intergenic regions are depicted in panel A,C,E,G. Histograms evidencing the location of motifs up to 500 nucleotides from the start codon are depicted in panels B,D,F,H. Motifs are separated by the temporal class of the respective genes. [file Image_8.TIF]

**MDS Plot: infection\_time**

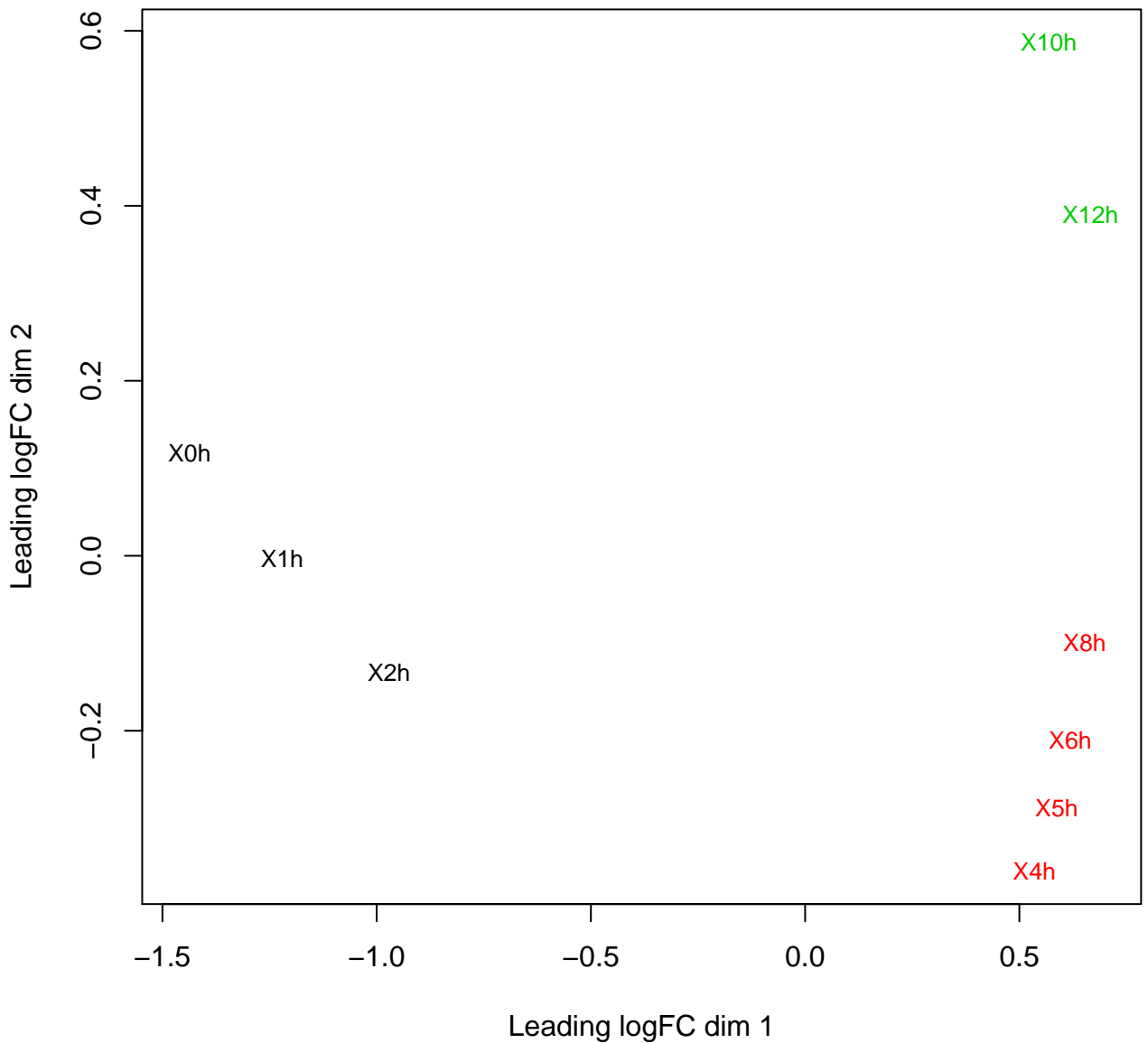

Supplement: FIGURE S10 — Multi-dimensional scaling of Acanthamoeba castellanii Neff RNA-seq read counts showing the three temporal groups: - early group in black with 0, 1, and 2 h infection time (0 h corresponds to 30 min of infection due to virus adsorption period); intermediate group (red) with 4, 5, 6, and 8 h infection time; and late group (green) 10 and 12 h infection time. [file Image_10.PDF]

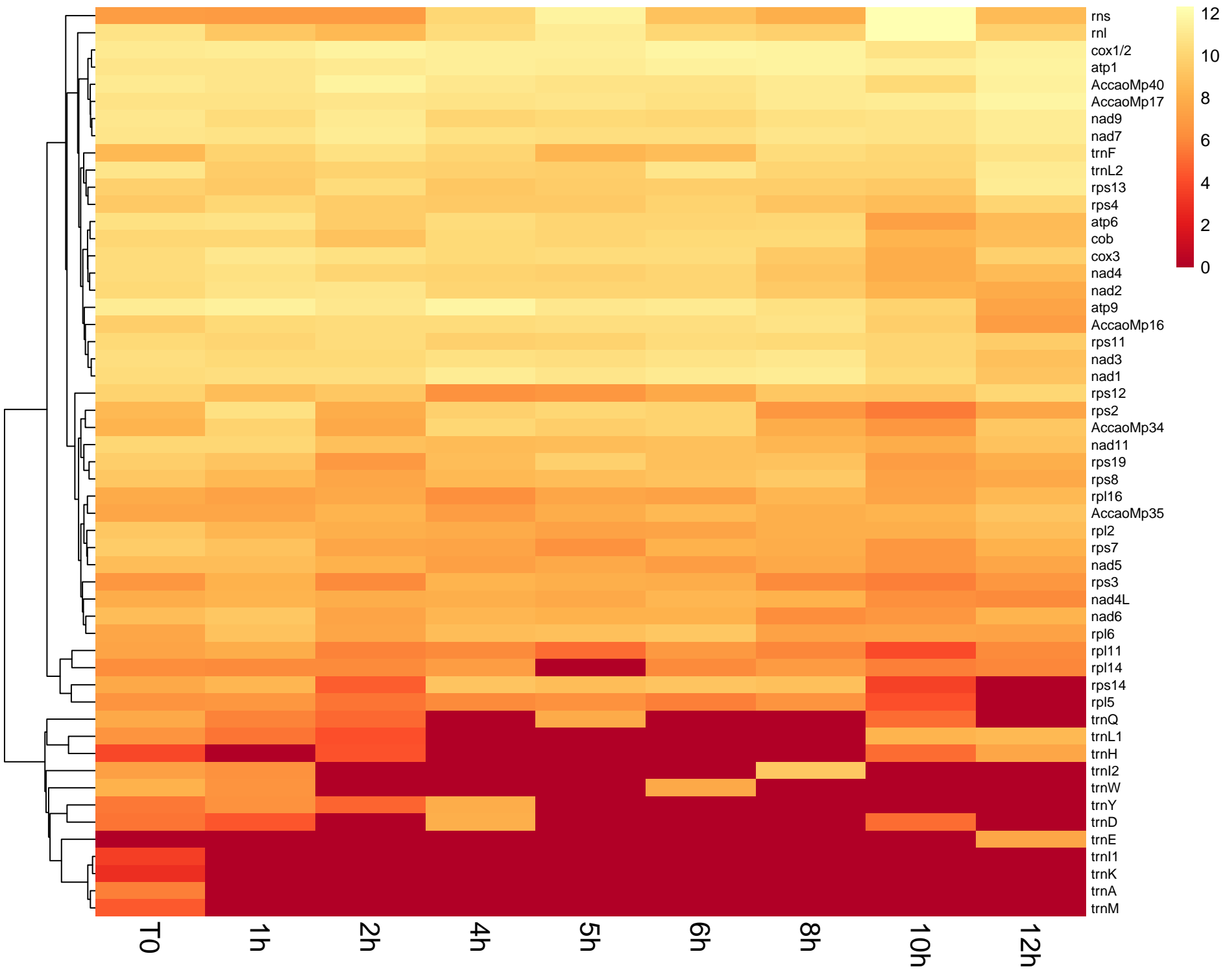

Supplement: FIGURE S12 — Acanthamoeba castellanii neff mitochondrion gene expression classes. Heatmap of mitochondrial gene expression profiles. Rows correspond to the 53 mitochondrial genes and columns to the 9 infection time points (0 h corresponds to 30 min of infection due to virus adsorption period). Expression profiles are clustered using Euclidean distance and average linkage of log-transformed normalized expression counts. A dendrogram of the clustering is shown on the left. Genes could be partitioned into three main group, “Genes with high expression levels” (top), “Genes with intermediate expression level” (center), and “Genes with low expression levels” (bottom). Expression levels are displayed from Red (low expression) to yellow (high expression). The function of each gene related to their level of expression pattern can be found in Supplementary Data Sheet S1. [file Image_12.PDF]
