# Supplementary material for: Analysis of a Marseillevirus Transcriptome Reveals Temporal Gene Expression Profile and Host Transcriptional Shift
Source: Front Microbiol. 2020 Apr 14;11:651. doi: 10.3389/fmicb.2020.00651 (PMC7192143; doi:10.3389/fmicb.2020.00651)
Supplement: FIGURE S9 — Promoter motifs found in Marseillevirus genome using BaMMmotif server. The motifs are presented from the most to the less representative. AvRec (Average Recall) - To avoid false predictions, the BaMM software determines true and false positive ratios (sensitivity and specificity) for each predicted promoter, a mean model recall averaged over range precision from 0 to 1 (R = TP/FP); estimates the false discovery rate (FDR) and then compute the log10R-values as a quality measure. Frac. occurrence (Fraction of sequences with motif) - Shows the occurrence (from 0 to 1) of the motif in the analyzed sequences. [file Image_9.PDF]

#	Forward strand	Complement strand	AvRec	Frac. occurrence
1	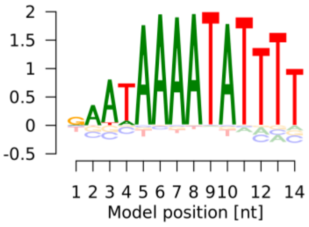	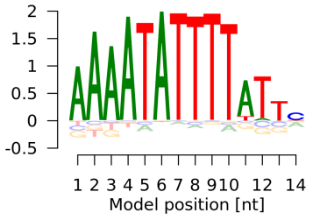	0.844	0.629
2	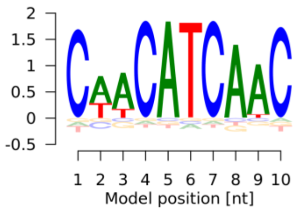	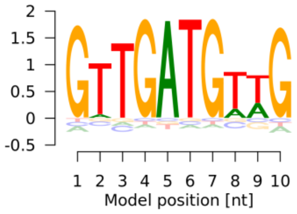	0.654	0.203
3	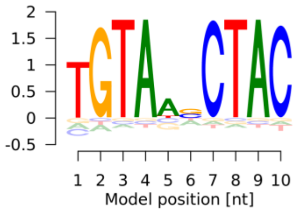	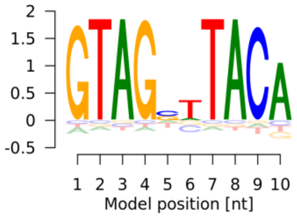	0.884	0.058
4	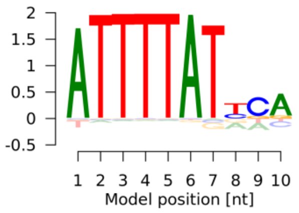	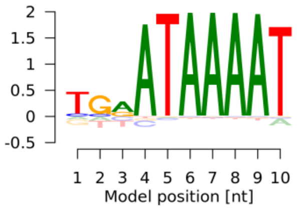	0.553	0.457
