# Supplementary material for: Analysis of a Marseillevirus Transcriptome Reveals Temporal Gene Expression Profile and Host Transcriptional Shift
Source: Front Microbiol. 2020 Apr 14;11:651. doi: 10.3389/fmicb.2020.00651 (PMC7192143; doi:10.3389/fmicb.2020.00651)
Supplement: FIGURE S11 — Expression levels of up and down regulated genes of Acanthamoeba castellanii during the course of infection by Marseillevirus. The function of each gene is depicted in Table 1. [file Image_11.PDF]

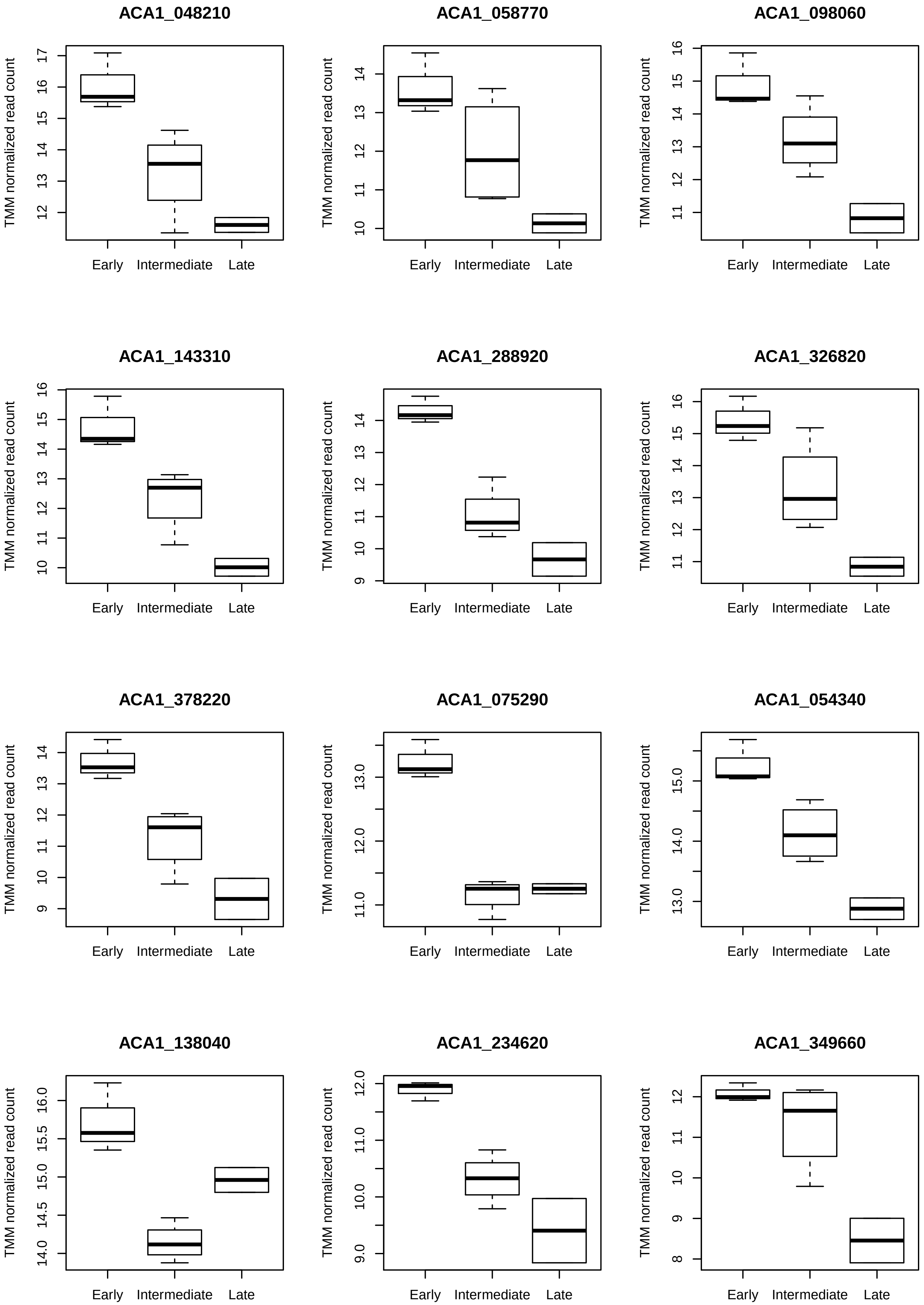

ACA1_048210
ACA1_058770
ACA1_098060
ACA1_143310
ACA1_288920
ACA1_326820
ACA1_378220
ACA1_075290
ACA1_054340
ACA1_138040
ACA1_234620
ACA1_349660
TMM normalized read count
Early
Intermediate
Late

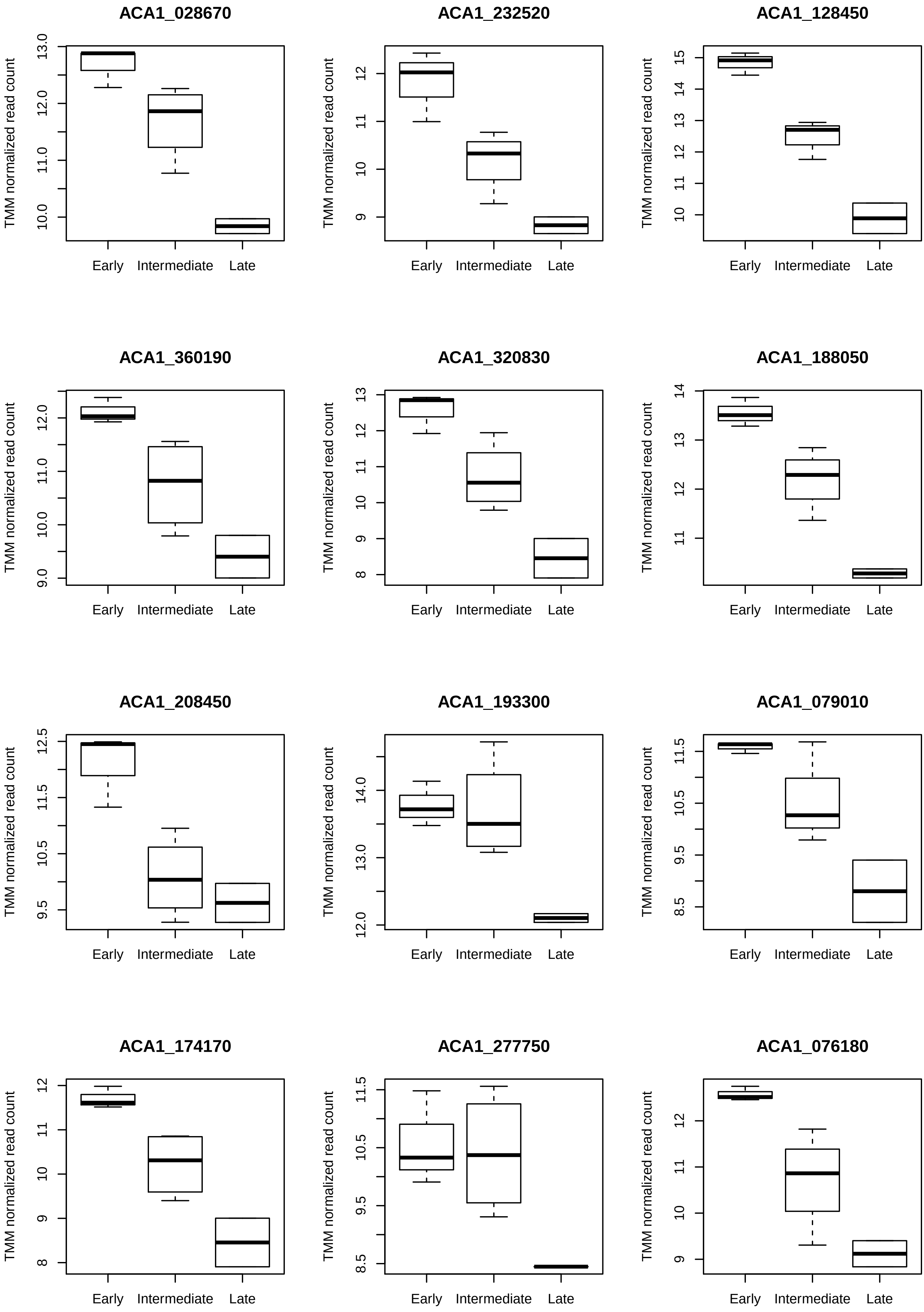

ACA1_028670
TMM normalized read count
10.0
11.0
12.0
13.0
Early
Intermediate
Late
ACA1_232520
TMM normalized read count
9
10
11
12
Early
Intermediate
Late
ACA1_128450
TMM normalized read count
10
11
12
13
14
15
Early
Intermediate
Late
ACA1_360190
TMM normalized read count
9.0
10.0
11.0
12.0
Early
Intermediate
Late
ACA1_320830
TMM normalized read count
8
9
10
11
12
13
Early
Intermediate
Late
ACA1_188050
TMM normalized read count
11
12
13
14
Early
Intermediate
Late
ACA1_208450
TMM normalized read count
9.5
10.5
11.5
12.5
Early
Intermediate
Late
ACA1_193300
TMM normalized read count
12.0
13.0
14.0
Early
Intermediate
Late
ACA1_079010
TMM normalized read count
8.5
9.5
10.5
11.5
Early
Intermediate
Late
ACA1_174170
TMM normalized read count
8
9
10
11
12
Early
Intermediate
Late
ACA1_277750
TMM normalized read count
8.5
9.5
10.5
11.5
Early
Intermediate
Late
ACA1_076180
TMM normalized read count
9
10
11
12
Early
Intermediate
Late

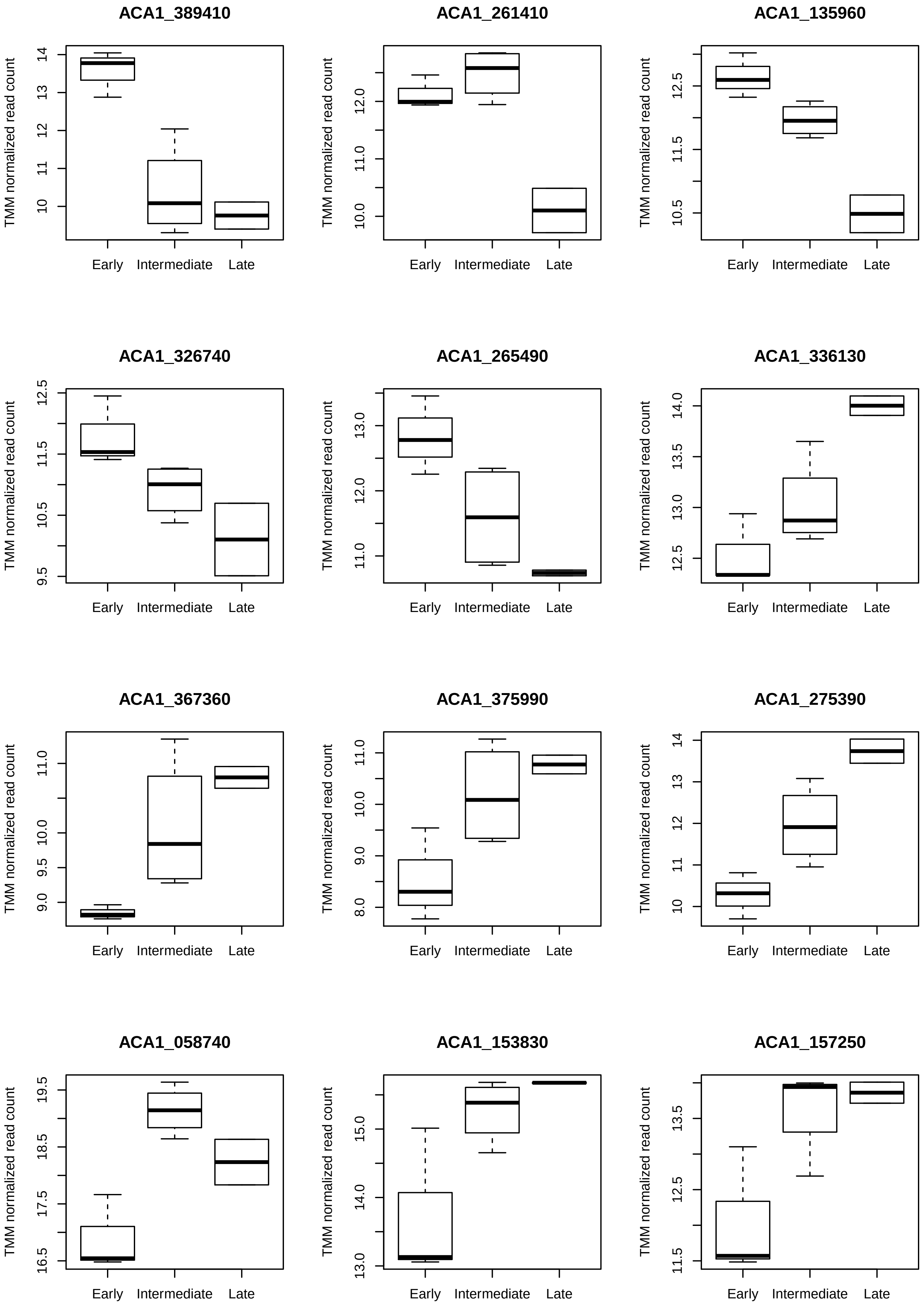
ACA1_389410
TMM normalized read count
10
11
12
13
14
Early
Intermediate
Late
ACA1_261410
TMM normalized read count
10.0
11.0
12.0
Early
Intermediate
Late
ACA1_135960
TMM normalized read count
10.5
11.5
12.5
Early
Intermediate
Late
ACA1_326740
TMM normalized read count
9.5
10.5
11.5
12.5
Early
Intermediate
Late
ACA1_265490
TMM normalized read count
11.0
12.0
13.0
Early
Intermediate
Late
ACA1_336130
TMM normalized read count
12.5
13.0
13.5
14.0
Early
Intermediate
Late
ACA1_367360
TMM normalized read count
9.0
9.5
10.0
11.0
Early
Intermediate
Late
ACA1_375990
TMM normalized read count
8.0
9.0
10.0
11.0
Early
Intermediate
Late
ACA1_275390
TMM normalized read count
10
11
12
13
14
Early
Intermediate
Late
ACA1_058740
TMM normalized read count
16.5
17.5
18.5
19.5
Early
Intermediate
Late
ACA1_153830
TMM normalized read count
13.0
14.0
15.0
Early
Intermediate
Late
ACA1_157250
TMM normalized read count
11.5
12.5
13.5
Early
Intermediate
Late

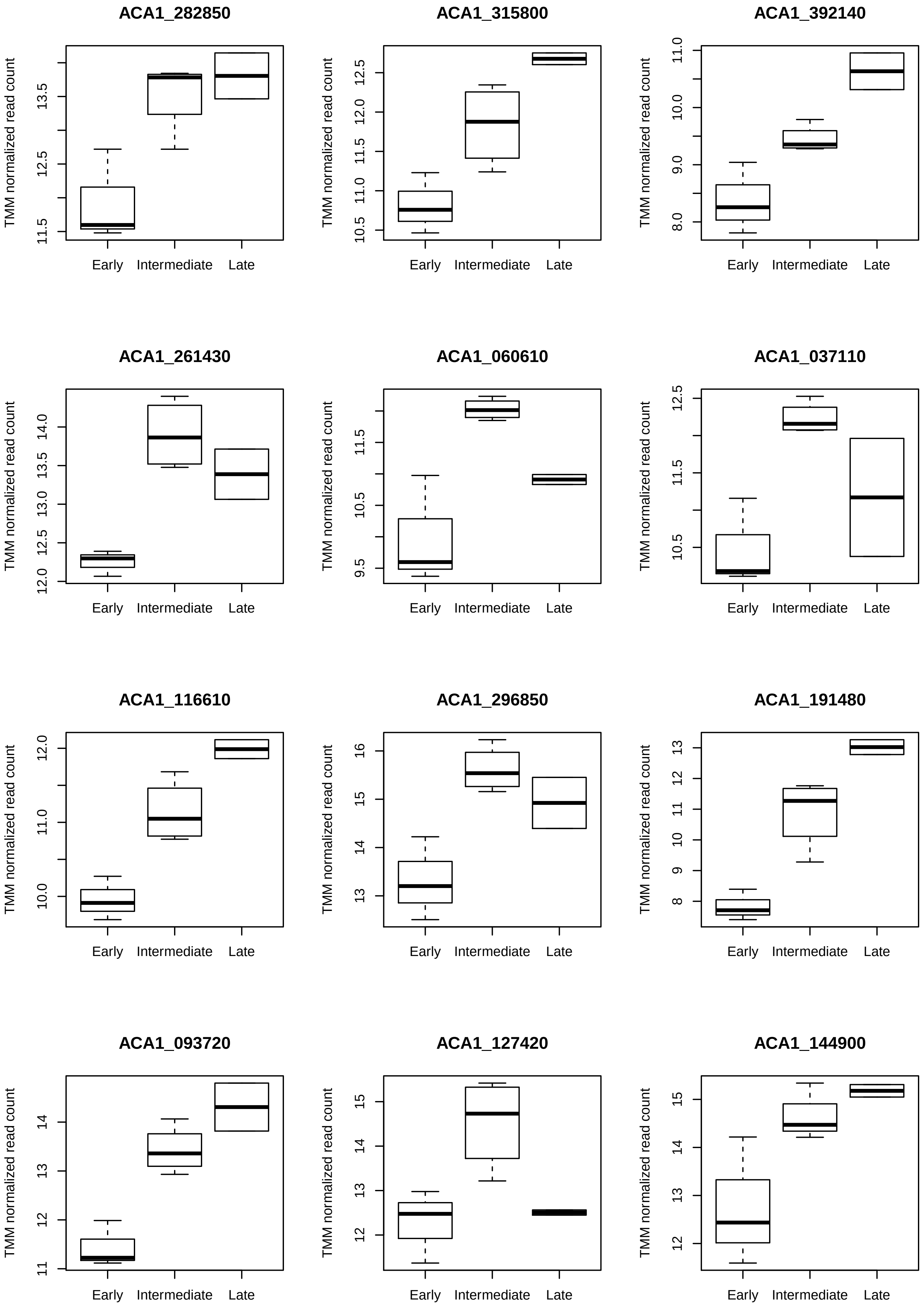
ACA1_282850
TMM normalized read count
11.5
12.5
13.5
Early
Intermediate
Late
ACA1_315800
TMM normalized read count
10.5
11.0
11.5
12.0
12.5
Early
Intermediate
Late
ACA1_392140
TMM normalized read count
8.0
9.0
10.0
11.0
Early
Intermediate
Late
ACA1_261430
TMM normalized read count
12.0
12.5
13.0
13.5
14.0
Early
Intermediate
Late
ACA1_060610
TMM normalized read count
9.5
10.5
11.5
Early
Intermediate
Late
ACA1_037110
TMM normalized read count
10.5
11.5
12.5
Early
Intermediate
Late
ACA1_116610
TMM normalized read count
10.0
11.0
12.0
Early
Intermediate
Late
ACA1_296850
TMM normalized read count
13
14
15
16
Early
Intermediate
Late
ACA1_191480
TMM normalized read count
8
9
10
11
12
13
Early
Intermediate
Late
ACA1_093720
TMM normalized read count
11
12
13
14
Early
Intermediate
Late
ACA1_127420
TMM normalized read count
12
13
14
15
Early
Intermediate
Late
ACA1_144900
TMM normalized read count
12
13
14
15
Early
Intermediate
Late
