# Supplementary material for: Analysis of a Marseillevirus Transcriptome Reveals Temporal Gene Expression Profile and Host Transcriptional Shift
Source: Front Microbiol. 2020 Apr 14;11:651. doi: 10.3389/fmicb.2020.00651 (PMC7192143; doi:10.3389/fmicb.2020.00651)
Supplement: TABLE S1 — Genes and primers used in RT-qPCR reactions. [file Table_1.PDF]

Supplementary table 1: Genes and primers used in RT-qPCR reactions.

Gene	Forward primer (5’-3’)	Reverse primer (5’-3’)	Tm (ºC)
MAR_ORF147	agc cat tgg aag agc aga gg	tgc ctt ccc ata tcg ttc gc	60
MAR_ORF342	ggt ttt gtg gac ttg gcg ac	cct ttt tga tgg cac gca ca	60
MAR_ORF354	caa cac aac cgg acc aga ga	ggg gct cgc ttt ttc ttt cc	60
MAR_ORF375	ggg gag gtc agt tgt tct cg	cca gag cag tct cgg tat cc	60
MAR_ORF412	cct gtg aga tgg cag gag tc	cct tcc tct tcc ccg tca aa	60
MAR_ORF421	gca aga gcc cca aaa aga gg	ccg tgt tgt acg gaa tca gc	60
