# Supplementary material for: Analysis of a Marseillevirus Transcriptome Reveals Temporal Gene Expression Profile and Host Transcriptional Shift
Source: Front Microbiol. 2020 Apr 14;11:651. doi: 10.3389/fmicb.2020.00651 (PMC7192143; doi:10.3389/fmicb.2020.00651)
Supplement: TABLE S3 — The top 20 most highly expressed annotated Marseillevirus genes. The expression data are represented as fractions Top 20 most expressed annotated genes. Expression level [(number of normalized reads of a time/total number of normalized reads of the gene) × 100] of each gene is exhibited. The raw data in Transcript Per Million reads are depicted in Table 1 (0 h corresponds to 30 min of infection due to virus adsorption period). N: no; Y: yes; E: early; I: intermediate; L: late. [file Table_3.DOC]

**Supplementary table 3: Most expressed genes**

Top 20 most expressed annotated genes. Expression level [(number of normalized reads of a time/total number of normalized reads of the gene)*100] of each gene is exhibited. The raw data in Transcript Per Million reads are depicted in Table 1.

| **Gene** | **Presence of promoter** | **Expression pattern** | **Predicted function** | **0h** | **1h** | **2h** | **4h** | **5h** | **6h** | **8h** | **10h** | **12h** |
| --- | --- | --- | --- | --- | --- | --- | --- | --- | --- | --- | --- | --- |
| MAR_ORF300 | N | L | - | 0,42 | 0,01 | 0,02 | 11,50 | 15,41 | 15,79 | 17,52 | 21,09 | 18,23 |
| MAR_ORF391 | Y | L | multiple zinc ribbon protein | 0,56 | 3,11 | 8,56 | 16,12 | 15,62 | 13,62 | 12,40 | 14,19 | 15,82 |
| MAR_ORF317 | N | L | - | 0,49 | 0,06 | 0,01 | 13,17 | 14,35 | 14,24 | 19,67 | 13,05 | 24,95 |
| MAR_ORF342 | Y | L | major capsid protein | 0,23 | 0,02 | 0,01 | 22,76 | 17,03 | 14,86 | 24,76 | 8,87 | 11,46 |
| MAR_ORF384 | N | L | - | 0,35 | 0,05 | 0,01 | 15,02 | 13,86 | 16,44 | 19,48 | 21,23 | 13,56 |
| MAR_ORF370 | N | L | - | 0,54 | 0,13 | 0,39 | 7,12 | 11,93 | 11,24 | 16,48 | 37,24 | 14,93 |
| MAR_ORF219 | Y | L | - | 0,35 | 0,05 | 5,66 | 37,65 | 21,00 | 9,83 | 8,97 | 7,80 | 8,69 |
| MAR_ORF029 | N | I | - | 0,87 | 11,35 | 28,60 | 12,98 | 9,07 | 8,33 | 11,55 | 6,68 | 10,58 |
| MAR_ORF305 | Y | L | - | 0,20 | 0,02 | 0,01 | 19,10 | 20,91 | 23,13 | 15,19 | 8,05 | 13,38 |
| MAR_ORF413 | Y | L | histone H3 | 0,30 | 0,03 | 0,04 | 9,40 | 20,78 | 24,12 | 17,44 | 12,97 | 14,93 |
| MAR_ORF109 | Y | L | - | 0,88 | 0,25 | 23,49 | 28,14 | 17,01 | 8,89 | 5,53 | 5,59 | 10,22 |
| MAR_ORF421 | N | L | - | 0,20 | 0,06 | 1,89 | 17,09 | 16,51 | 20,42 | 20,42 | 8,87 | 14,54 |
| MAR_ORF412 | N | E | - | 53,58 | 39,59 | 5,82 | 0,19 | 0,10 | 0,19 | 0,13 | 0,20 | 0,19 |
| MAR_ORF026 | Y | L | - | 0,40 | 0,18 | 0,17 | 6,77 | 15,50 | 19,40 | 15,60 | 14,71 | 27,29 |
| MAR_ORF193 | Y | L | zinc finger protein | 0,39 | 0,08 | 2,40 | 14,10 | 17,05 | 15,08 | 16,65 | 16,06 | 18,20 |
| MAR_ORF021 | Y | L | - | 0,73 | 0,08 | 0,02 | 8,96 | 16,84 | 20,23 | 19,75 | 15,80 | 17,60 |
| MAR_ORF389 | Y | E | - | 28,87 | 45,73 | 22,24 | 0,54 | 0,43 | 0,56 | 0,41 | 0,77 | 0,46 |
| MAR_ORF250 | N | L | - | 0,21 | 0,24 | 0,10 | 16,42 | 21,78 | 22,47 | 17,33 | 10,24 | 11,22 |
| MAR_ORF014 | N | E | - | 72,47 | 24,33 | 2,79 | 0,11 | 0,09 | 0,02 | 0,06 | 0,06 | 0,07 |
| MAR_ORF147 | N | E | - | 53,01 | 43,42 | 2,88 | 0,17 | 0,11 | 0,14 | 0,10 | 0,09 | 0,07 |

N: no; Y: yes; E: early; I: intermediate; L: late.
