# Supplementary material for: Analysis of a Marseillevirus Transcriptome Reveals Temporal Gene Expression Profile and Host Transcriptional Shift
Source: Front Microbiol. 2020 Apr 14;11:651. doi: 10.3389/fmicb.2020.00651 (PMC7192143; doi:10.3389/fmicb.2020.00651)
Supplement: TABLE S6 — Promoter motif search using MEME software considering sequence size of 8∼10 and 5∼15. Motif sequences are ordered according to statistical e-value. [file Table_6.DOC]

**Supplementary table 6:** Promoter motif search using MEME software considering sequence size of 8~10 and 5~15. Motif sequences are ordered according to statistical e-value.

| # | 8 ~ 10 nucleotides | 5 ~ 15 nucleotides |
| --- | --- | --- |
| 1 | AAAATATT width = 8 sites = 890 E-value = 8.0e-143 | AAAATATTTTWT width = 12 sites = 979 E-value = 5.9e-167 |
| 2 | TTYTCTTY width = 8 sites = 1000 E-value = 5.7e-142 | CVARARRVAVRARAR width = 15 sites = 1499 E-value = 1.4e-148 |
| 3 | TTYTSGAR width = 8 sites = 1000 E-value = 1.0e-072 | YTCTTTB width = 7 sites = 2010 E-value = 3.4e-114 |
| 4 | CTCTYTYY width = 8 sites = 1000 E-value = 1.8e-087 | TYBVARARA width = 9 sites = 708 E-value = 1.2e-045 |
| 5 | CAAARRAR width = 8 sites = 1000 E-value = 3.7e-066 | VVRARNYTYTYS width = 12 sites = 1370 E-value = 3.8e-015 |
| 6 | GRRARGAA width = 8 sites = 953 E-value = 1.6e-036 | TTTTTSTGG width = 9 sites = 147 E-value = 2.7e-005 |
| 7 | SAGARAAA width = 8 sites = 770 E-value = 2.7e-018 | YTYTYYSTYCWTCTC width = 15 sites = 90 E-value = 7.5e-003 |
