# Supplementary material for: Analysis of a Marseillevirus Transcriptome Reveals Temporal Gene Expression Profile and Host Transcriptional Shift
Source: Front Microbiol. 2020 Apr 14;11:651. doi: 10.3389/fmicb.2020.00651 (PMC7192143; doi:10.3389/fmicb.2020.00651)
Supplement: TABLE S7 — Statistical analysis of promoter motifs combination in association to different temporal classes of genes. [file Table_7.PDF]

ANOVA Motif 1

	SS	df	F	p	partial η^2
Groups	2.904	2	0.420	0.658	0.002
Motif 1 x Motif 2	4.177	1	1.207	0.273	0.003
Motif 1 x Motif 3	6.351	1	1.836	0.176	0.005
Motif 1 x Motif 4	9.403	1	2.718	0.100	0.008
Residuals	1235.041	357			

ANOVA Motif 2

	SS	df	F	p	partial η^2
Groups	13.987	2	1.038	0.355	0.006
Motif 2 x Motif 1	4.907	1	0.729	0.394	0.002
Motif 2 x Motif 3	1.822	1	0.271	0.603	0.001
Motif 2 x Motif 4	6.123	1	0.909	0.341	0.003
Residuals	2404.608	357			

ANOVA Motif 3

	SS	df	F	p	partial η^2
Groups	0.839	2	0.045	0.956	0
Motif 3 x Motif 1	0.015	1	0.002	0.969	0
Motif 3 x Motif 2	0.209	1	0.022	0.881	0
Motif 3 x Motif 4	1.517	1	0.161	0.688	0
Residuals	3352.747	357			

ANOVA Motif 4

	SS	df	F	p	partial η^2
Groups	14.265	2	1.649	0.194	0.009
Motif 4 x Motif 1	3.528	1	0.816	0.367	0.002
Motif 4 x Motif 2	1.570	1	0.363	0.547	0.001
Motif 4 x Motif 3	7.512	1	1.737	0.188	0.005
Residuals	1544.167	357			
