# Supplementary material for: Analysis of a Marseillevirus Transcriptome Reveals Temporal Gene Expression Profile and Host Transcriptional Shift
Source: Front Microbiol. 2020 Apr 14;11:651. doi: 10.3389/fmicb.2020.00651 (PMC7192143; doi:10.3389/fmicb.2020.00651)
Supplement: TABLE S8 — Assignment of RNA-seq reads according to their origin across samples. The absolute number of generated RNA-seq reads and reads mapping to the viral amoebal nuclear and mitochondrial genome is presented as well as the percentage of each fraction from the assigned reads. [file Table_8.DOC]

**Supplementary table 8:** Assignment of RNA-seq reads according to their origin across samples. The absolute number of generated RNA-seq reads and reads mapping to the viral, amoebal nuclear and mitochondrial genome is presented as well as the percentage of each fraction from the assigned reads.

| **Dataset** | **Raw reads count** | **Reads mapping to Marseillevirus marseillevirus genome** | | **Reads mapping to *Acanthamoeba castellanii* nuclear genome** | | **Reads mapping to *Acanthamoeba castellanii* mitochondrial genome** | | **Total assigned reads** |
| --- | --- | --- | --- | --- | --- | --- | --- | --- |
| **0h** | 12 175 245 | 137 540 | 2% | 3 730 562 | 66% | 1 822 740 | 32% | 5 690 842 |
| **1h** | 4 451 972 | 167 123 | 17% | 636 875 | 64% | 223 460 | 22% | 1 027 458 |
| **2h** | 5 799 822 | 2 909 752 | 78% | 574 782 | 15% | 260 116 | 7% | 3 744 650 |
| **4h** | 543 207 | 251 511 | 92% | 16 125 | 6% | 5 270 | 2% | 272 906 |
| **5h** | 681 747 | 256 230 | 91% | 17 019 | 6% | 8 514 | 3% | 281 763 |
| **6h** | 613 367 | 254 229 | 90% | 19 713 | 7% | 8 589 | 3% | 282 531 |
| **8h** | 383 551 | 189 780 | 93% | 11 824 | 6% | 3 209 | 2% | 204 813 |
| **10h** | 8 401 851 | 4 551 047 | 94% | 177 967 | 4% | 133 470 | 3% | 4 862 484 |
| **12h** | 1 923 942 | 1 087 013 | 95% | 54 849 | 5% | 8 556 | 1% | 1 150 418 |
| **Total** | **34 974 704** | **9 804 225** | **56%** | **5 239 716** | **30%** | **2 473 924** | 14% | **17 517 865** |
